# Brachyceran Diptera (Insecta) in Cretaceous ambers, Part IV, Significant New Orthorrhaphous Taxa

**DOI:** 10.3897/zookeys.148.1809

**Published:** 2011-11-21

**Authors:** David A. Grimaldi, Antonio Arillo, Jeffrey M. Cumming, Martin Hauser

**Affiliations:** 1 Division of Invertebrate Zoology, American Museum of Natural History, New York, New York, 10024, USA; 2 Departamento de Zoología y Antropología Física (Entomología), Facultad de Biología, Universidad Complutense, E-28040, Madrid, Spain;; 3 Canadian National Collection of Insects, Arachnids, and Nematodes, Agriculture and Agri-Food Canada, K.W. Neatby Building, 960 Carling Ave., Ottawa Canada K1A OC6; 4 California Department of Food and Agriculture, Plant Pest Diagnostics Branch, 3294 Meadowview Rd., Sacramento, California 95832–1448 USA

**Keywords:** amber, fossils, flies, Lebanon, Myanmar, New Jersey, Spain

## Abstract

Thirteen species of basal Brachycera (11 described as new) are reported, belonging to nine families and three infraorders. They are preserved in amber from the Early Cretaceous (Neocomian) of Lebanon, Albian of northern Spain, upper Albian to lower Cenomanian of northern Myanmar, and Late Cretaceous of New Jersey USA (Turonian) and Alberta, Canada (Campanian). Taxa are as follows, with significance as noted: In Stratiomyomorpha: Stratiomyidae (*Cretaceogaster pygmaeus* Teskey [2 new specimens in Canadian amber], *Lysistrata emerita* Grimaldi & Arillo, **gen. et sp. n.** [stem-group species of the family in Spanish amber]), and Xylomyidae (*Cretoxyla azari* Grimaldi & Cumming, **gen. et sp. n.** [in Lebanese amber], and an undescribed species from Spain). In Tabanomorpha: Tabanidae (*Cratotabanus newjerseyensis* Grimaldi, **sp. n**., in New Jersey amber). In Muscomorpha: Acroceridae (*Schlingeromyia minuta* Grimaldi & Hauser, **gen. et sp. n.** and *Burmacyrtus rusmithi* Grimaldi & Hauser **gen. et** **sp. n.**, in Burmese amber, the only definitive species of the family from the Cretaceous); Mythicomyiidae (*Microburmyia analvena* Grimaldi & Cumming **gen. et sp. n.** and *Microburmyia veanalvena* Grimaldi & Cumming, **sp. n.**, stem-group species of the family, both in Burmese amber); Apsilocephalidae or near (therevoid family-group) (*Kumaromyia burmitica* Grimaldi & Hauser, **gen. et sp. n**. [in Burmese amber]); Apystomyiidae (*Hilarimorphites burmanica* Grimaldi & Cumming, **sp. n.** [in Burmese amber], whose closest relatives are from the Late Jurassic of Kazachstan, the Late Cretaceous of New Jersey, and Recent of California). Lastly, two species belonging to families incertae sedis, both in Burmese amber: Tethepomyiidae (*Tethepomyia zigrasi* Grimaldi & Arillo **sp. n.**, the aculeate oviscapt of which indicates this family was probably parasitoidal and related to Eremochaetidae); and unplaced to family is *Myanmyia asteiformia* Grimaldi, **gen. et sp. n.**, a minute fly with highly reduced venation. These new taxa significantly expand the Mesozoic fossil record of rare and phylogenetically significant taxa of lower Brachycera.

## Introduction

This is the fourth paper in a series devoted to the Cretaceous record of brachyceran flies preserved in amber, the original work being a treatment of orthorrhaphans and Cyclorrhapha (Grimaldi and Cumming 1999), and the second and third being treatments of the enigmatic families Tethepomyiidae (Grimaldi and Arillo 2008) and Chimeromyiidae (Grimaldi et al. 2009). The present paper deals specifically with additional records and taxa of orthorrhaphan (“basal”) Brachycera, while separate papers will deal with the empidoids and Cyclorrhapha. Rhagionidae will also require separate study, given their confusing Mesozoic diversity and relationships. Updates to the original 1999 monograph are necessary since additional Brachycera have been uncovered in all of the major Cretaceous amber deposits dealt with then (i.e., New Jersey, western Canada, Lebanon), but, most importantly, diverse Brachycera have been discovered in amber from the Early Cretaceous (Albian) of northern Spain (Alonso et al. 2000; Delclòs et al. 2007; Peñalver and Delclòs 2010) and the Late Albian to Early Cenomanian of northern Myanmar (Grimaldi et al. 2002; Cruikshank and Ko 2003).

## Materials and methods

Specimens were prepared according to the protocols described in Nascimbene and Silverstein (2000) and, for the Spanish amber, in Corral et al. (1999). After rough pieces were screened for inclusions, preliminary preparation to view the inclusion was done by grinding and polishing one or more flat surfaces onto the piece using emory papers of decreasing grit sizes on a wet flat lapidary wheel. Then each piece was embedded in epoxy under vacuum in order to impregnate cracks within the amber, which improved transparency and strengthened the piece for trimming (using a water-fed diamond saw with a very thin blade) and more grinding and polishing. Eventually, depending on the size and number of inclusions, the preparations were several millimeters in thickness, such that it could be mounted between a microscope slide and coverslip using a drop of glycerin on each of the two opposing surfaces. The inclusions were examined with a stereomicroscope and with compound microscopy at 40 – 400x using both reflected and transmitted light.

**Collection repositories of specimens are the following:**

AMNH American Museum of Natural History, Entomology Section, New York.

AZ Azar Collection, presently housed in Musée national d’Histoire naturelle, Paris.

KU University of Kansas Division of Entomology, Natural History Museum, Lawrence.

MCNA Museo de Ciencias Naturales, Álava, Spain.

NHML Natural History Museum, London.

RTMP Royal Tyrrell Museum of Palaeontology, Drumheller, Alberta, Canada.

It is a pleasure for the senior author to dedicate this paper to Kumar Krishna, world authority on the Isoptera, close colleague and friend.

## Infraorder Stratiomyomorpha

This lineage comprises three living families, the Xylomyiidae (cosmopolitan; approximately 134 species in four genera), Stratiomyidae (cosmopolitan; 2651 species in 375 genera as of the year 2000 [Woodley 2001]), and Pantophthalmidae (Neotropical; approximately 20 species of very large flies in two genera). Xylomyids and stratiomyids are closely related based on the shortened vein R_1_, Rs branching off of R distally, and the stem of Rs very short, as well as the larval cuticle with calcareous “warts” and pupariation taking place within the last larval cuticle. Pantophthalmids appear to be the sister group to the other two families (Sinclair et al. 1994; Yeates 2002; Wiegmann et al. 2011). Pantophthalmids have no fossil record, and the other two families have very limited Mesozoic fossil records, reviewed below. A fourth family of the infraorder is the extinct Zhangsolvidae, originally erected by Nagatomi and Yang (1998) for *Zhangsolva cupressa* from the Early Cretaceous Laiyang Formation in China (Zhang et al. 1993). The original description of *Zhangsolva* included obvious errors, and the antenna probably has no more than eight flagellomeres (the groundplan of Brachycera); some artifacts were also reported as venational features in the original report. There are two well preserved zhangsolvid species in Early Cretaceous limestone from the Crato Formation of Brazil, *Cratomyia macrorrhyncha* (Mazzarolo & Amorim, 2000), and *Cratomyoides cretacicus* Wilkommen (in Martill et al. 2007). Both of these species have a long, jutting proboscis and a venation very similar to that *Zhangsolva*; *Cratomyoides* is separated from *Cratomyia* on the basis of minor features and probably should be synonymized with the latter genus. Based on the long proboscis, hovering-type of venation, and phylogenetic position, the three species of Zhangsolvidae probably fed from flowers (Grimaldi and Engel 2005).

### Family Stratiomyidae

#### Genus Cretaceogaster

http://species-id.net/wiki/Cretaceogaster

Cretaceogaster Teskey 1971: 1660. Type species: *Cretaceogaster pygmaeus*Teskey 1971: 1660; Woodley 1986 (redescription, placement); Grimaldi and Cumming 1999: 17–19 (redescription, new specimens).
Cretaceogaster pygmaeus Teskey 1971: 1660.

##### Remarks.

We were able to study two additional specimens of this very primitive genus of stratiomyid, both in Canadian amber collected by Ted Pike from Grassy Lake, Alberta (Campanian) (Pike 1995), housed in the RTMP.

RTMP 96.9.1117: Amber is a typical clear, dark yellow with reddish flow lines; it also contains a small spider. The piece is a cylindrical runnel 12 × 4 × 2 mm, with the fly preserved near the middle, which was embedded in epoxy at the AMNH and trimmed to 9 × 13 × 4 mm (including epoxy) for better observation. The fly is laterally very flattened, especially the thorax, and is a male (though details of the genitalia are not observable). Unfortunately, the apex of the mid tibia cannot be observed in detail, so the apparent absence of tibial spurs is uncertain. Wing is slightly distended in length, but otherwise the venation is very similar to *Cretaceogaster pygmaeus*.

RTMP 96.9.1230: Fly is also preserved in a cylindrical runnel of amber, 7 × 3 (diam.) mm, and embedded in epoxy for careful trimming. The fly is lying at the rounded end of the runnel, with its dorsal surface against the surface of the flow. The thorax is partly decayed and wing venation is obscured. The antenna and mouthparts are visible in ventral view. Specimen is a male, but its genitalic details are also not observable. Mid tibia appears to have a small apical spur, contrary to the original description of the species but in agreement with Woodley (1986: 380).

#### Lysistrata

Grimaldi & Arillo gen. n.

urn:lsid:zoobank.org:act:C038F6C9-1BFF-49D2-A756-A6235259B6A9

http://species-id.net/wiki/Lysistrata

##### Diagnosis.

Antennal flagellum submoniliform, with approximately 7 short flagellomeres tapered in width apicad; articulation between basal 3 flagellomeres faint. Protibia lacking spurs; mesotibia with two short apical spurs (c. 50 µm length). Metatibia probably with one pair of short apical spurs. Vein Rs branches from R_1_ in the distal third of vein R. Stem of R_4+5_ straight, R_4_ curved basally, long and subparallel to R_5_. Cell d long and narrow, length approximately 3.5x the width; cell m_3_ absent.

##### Type species.

*Lysistrata emerita*, sp. n., by present designation.

##### Etymology.

From the Greek, Λυσιστράτη, meaning “army disbander”, after the comedy by Aristophanes and in reference to the common name for Stratiomyidae, or “soldier flies”. Feminine.

##### Discussion.

*Lysistrata* is clearly within the Stratiomyomorpha, and appears closely allied with Stratiomyidae on the basis of the radial branching. The presence of two minute spurs on the mesotibia, and probably a short pair on the metatibia is indicative of either Stratiomyidae or Xylomyidae. A few Recent stratiomyids have a minute apical spur on the mesotibia, whereas xylomyids have either a 0–2–2 or 0–2–1 tibial spur formula. Pantophthalmids have one or two spurs on the mesotibia only, but are distinct from the other two families by the longer branches of R_1 _and Rs.

The Recent and primitive genus *Parhadrestia* James (consisting of two species from Chile) shares some similarities with *Lysistrata*, both of them possessing a long R_4_ vein curved only at the base and with the main branch only slightly divergent from R_5_. The genus *Montsecia* Mostovski, 1999, preserved as a compression in Early Cretaceous (Barremian) limestone of Montsec, Lérida Province, Spain (originally and incorrectly placed in the subfamily Beridinae) also has the fork of R_4_+R_5_ quite long. This long fork may be a plesiomorphic feature, seen for example in Rhagionidae and Spaniidae.

*Lysistrata* differs plesiomorphically from *Parhadrestia* by the following: antenna multiarticulate; wing longer, narrower; R_2+3_ slightly longer and gradually sloped to C; apex of R_2+3_ not close to the apex of R_1_; R_5_ and M_1 _slightly divergent instead of parallel; M_1_, M_2_, and CuA_1_ not as divergent (a condition shared with *Montsecia*); cell d much longer, its length approximately 3× the width (vs. 2× the width in *Montsecia* and 1.5× the width in *Parhadrestia*; in most Recent stratiomyids cell d is quite small); CuA_2_ more sloped toward CuP (e.g., apex of cell cup acute, instead of truncate [similar to *Montsecia*], although an acute cell cup is considered apomorphic by Woodley [2001]). In *Montsecia* the base of M is weak, whereas it is well developed in *Lysistrata*. *Lysistrata* has two apomorphic features: small female abdominal segments 6 and 7, which telescope within the proximal ones (in the basal Recent subfamilies Parhadrestiinae, Chiromyzinae and Beridinae segments 6 and 7 are large [Woodley 2001]); also, vein M_3_ is lost. Loss of this vein occurs in all Parhadrestiinae and Pachygastrinae, and is frequently absent in Chiromyzinae and Beridinae (Woodley 2001). Absence of M_3_ may actually be a ground-plan feature of Stratiomyidae.

The oldest fossil stratiomyiid is *Montsecia martinezdelclosi* Mostovski (1999), from the same outcrop that yielded several larvae believed to be stratiomyiids (Whalley and Jarzembowski 1985). According to Mostovski (1999), several undescribed stratiomyiids are known from Jurassic and Cretaceous outcrops of Kazakhstan and Russia, although none has as yet been described. *Gigantoberis liaoningensis*, described as a stratiomyiid by Huang and Lin (2007) from the Early Cretaceous of Lianoning, China, was shown by Zhang (2009) not to belong to this family, which Huang acknowledges (pers. comm. to AA, 2010). The only other Cretaceous stratiomyiids are *Cretaceogaster pygmaeus* (Teskey 1971; Grimaldi and Cumming 1999; herein *vide supra*), an incomplete and undescribed species in Turonian-aged amber from New Jersey USA (Grimaldi and Cumming 1999), and the very well-preserved *Lysistrata emerita*, described below and which is very basal in the family. Diverse stratiomyiids belonging to modern subfamilies and genera, including undescribed species, occur in shales and amber from the Tertiary and were summarized in Evenhuis (1994).

#### Lysistrata emerita

Grimaldi & Arillo sp. n.

urn:lsid:zoobank.org:act:FD339789-7504-41CB-832A-23238FC6675A

http://species-id.net/wiki/Lysistrata emerita

Fig. 1

##### Diagnosis.

As for the genus.

##### Description.

Body length 5.75 mm. Head length 0.60 mm. Specimen well preserved, but only visible in lateral view. Head slight distorted, with right antenna slightly separated from base. Eyes bare, large, covering most of head; facets not differentiated. Ocellar triangle not visible. Antenna submoniliform, with approximately 7 short flagellomeres tapered in width distad (articulations between 3 basal flagellomeres faint, number of articles difficult to discern); length of antenna approximately equal to length of head; length of flagellum 3× that of scape + pedicel combined. Distal flagellomere distinctly longer and narrower than more basal ones. Palpi reduced, segmentation not discernable; labellum well developed. Thorax: Mesonotum short and compact, finely pilose dorsally, without macrosetae. Scutellum without spines. Surface of notum slightly metallic and foveolate. All legs preserved; protibia lacking spurs; mesotibia with two apical spurs (50 µm long); probably one short apical spur on metatibiae. Length of hind basitarsomere equal to that of tarsomeres 2–5. Wing length 3.05 mm, width 0.75 mm; hyaline, vein Sc straight, length approximately 0.45× wing length, complete. Lengths of costal section of wing between apices of R_2+3_ and R_1_ equal to that between R_1_ and Sc. R_2+3_ arising distant from r-m. R_4+5_ straight, R_4_ curved at base, long and subparallel to R_5_. Veins M_1_ and M_2_ separated at discal cell. Cell m_3_ absent, vein M_3_ either absent or fused to CuA_1_. Abdomen elongate; basal 4 or 5 segments large and wide; apical 5 segments narrow and telescoping. Cercus composed of 2 segments, basal segment longer than apical one.

**Figure 1.**
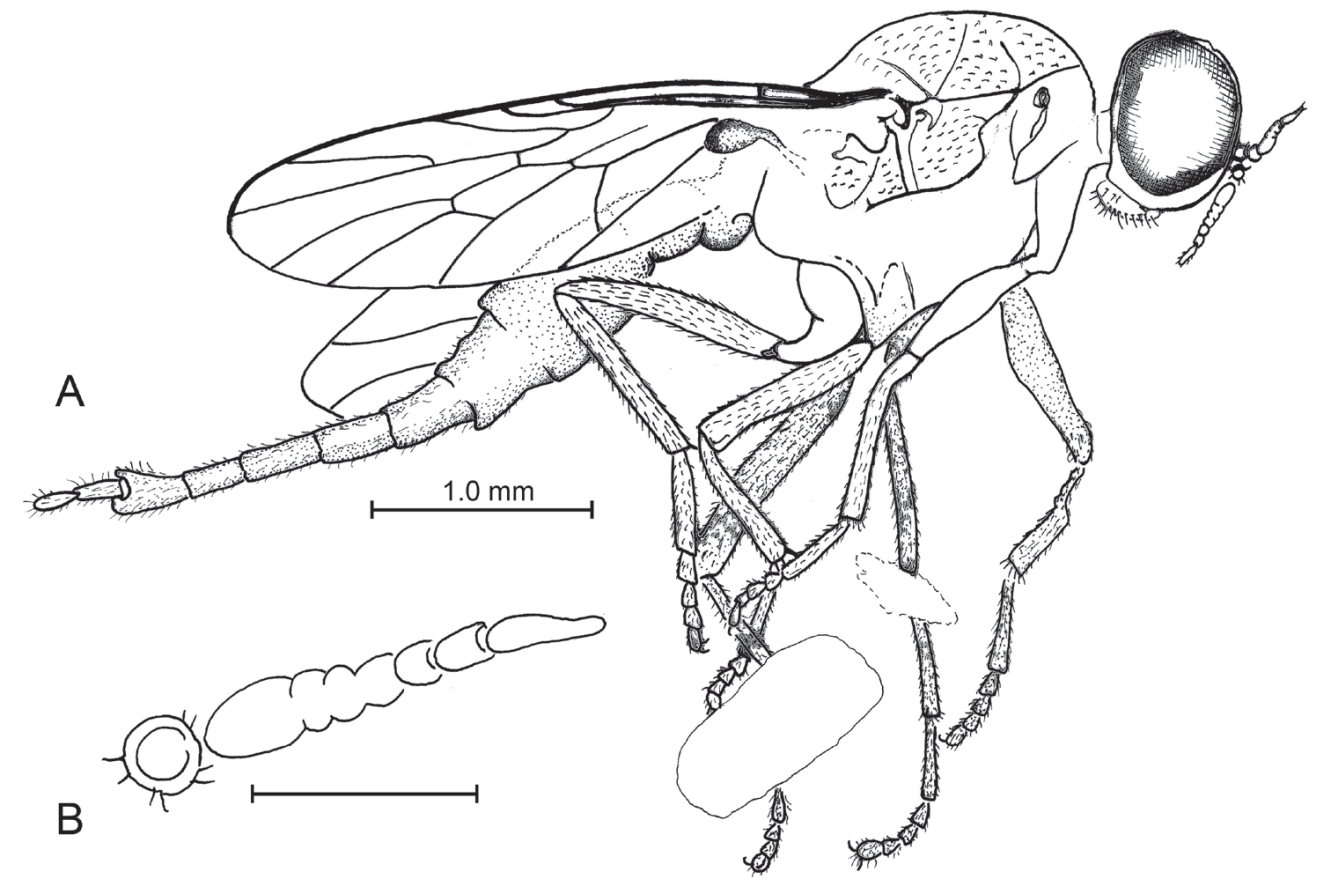
**a, b** *Lysistrata emerita* Grimaldi & Arillo, gen. et sp. n (Stratiomyidae) in Albian amber from Spain. Holotype, MCNA 12698 **a** lateral view **b** Antenna (scale bar 0.25 mm).

##### Type.

Holotype, female, MCNA 12698, SPAIN: Alava, Peñacerrada I (Moraza), Escucha Formation, Lower Cretaceous (Albian). Deposited in MCNA. Specimen is well preserved in a clear piece of amber 10 × 7 × 1.5 mm, partially missing the left side of the thorax and the left wing; the amber is embedded in epoxy 15 × 13 × 2 mm. An empidoid fly (Microphorinae) is present as a syninclusion.

##### Etymology.

From the Latin noun, *Emeritus*, a name given to retired Roman soldiers, used here in reference to this long-retired (i.e., extinct) species.

### Family Xylomyiidae

#### Cretoxyla

Grimaldi & Cumming gen. n.

urn:lsid:zoobank.org:act:EB99B611-9DD4-4B20-A808-02847EC2188D

http://species-id.net/wiki/Cretoxyla

##### Diagnosis.

Antenna thick, greatest width (in middle) 0.25x total length, with apparently 7 flagellomeres; protibia without apical spur; most distinctive features are in venation, which distinguishes this genus from other Mesozoic xylomyids by: vein M (separating cells br and bm) weak; cell m_3_ very small, width and length approximately half that of cell d (these are of equivalent size in other xylomyids, or m_3_ is slightly smaller), and, very distinctively, vein R_2+3_ is uniquely lost.

##### Type species.

*Cretoxyla azari* sp. n., by present designation.

##### Etymology.

From *Cret*aceous, and *Xylo*myiidae.

##### Discussion.

The closed wing cell m_3_ is a feature also seen in some Xylophagidae. *Cretoxyla*, however, apomorphically has no protibial spur (as in Stratiomyomorpha) and plesiomorphically does not have a reduced alula (a greatly reduced alula occurs in the Xylophagidae). The extent of vein C, particularly whether it extends only to the apex of M_1_ or M_2_ (Woodley 1986: 1377), unfortunately cannot be checked since the apical quarter of the wing is lost. Vein C is definitely not circumambient. Other features that are important for xylomyid relationships that cannot be observed in the incomplete fossil are the number of palpal segments (1 vs. 2), presence of denticles on the ventral surface of the hind femur (e.g., *Solva* Walker), and various male and female genitalic structures.

The oldest fossil record of Xylomyiidae is *?Xylomyia* [sic] *shcherbakovi* Mostovski from the Upper Jurassic (Karabastau Formation) of Kazakhastan (Mostovski 1999). Zhang and Zhang (1993) indicated that *Mesosolva* Hong and *Prosolva* Hong, also described as xylomyids from the Upper Jurassic of China, belong in another lower brachyceran family. Undescribed Cretaceous xylomyids are from the Upper Cretaceous amber of Siberia (Zherikhin and Sukacheva 1973), and an incomplete specimen of an undescribed species in Spanish amber (*vide infra*), so *Cretoxyla* is the oldest Cretaceous xylomyid (Early Cretaceous, Neocomian). Tertiary fossil xylomyids are *Solva inornata* Melander, 1949 and *Xylomya moratula* Cockerell, 1914 in late Eocene shale from Florissant, Colorado; and *Solva nana* Loew, 1850 in mid-Eocene Baltic amber.

#### Cretoxyla azari

Grimaldi & Cumming sp. n.

urn:lsid:zoobank.org:act:0B1DC31D-17E2-4756-BDB2-E24C5E066E05

http://species-id.net/wiki/Cretoxyla_azari

Fig. 2

##### Diagnosis.

As for genus.

##### Description.

*Head*: Largely preserved, visible in oblique dorsal and ventral views. Head slightly flattened dorsoventrally, wider than deep, but exact proportions unclear since head seems somewhat distorted. Eyes large, bare, facets not differentiated; dorsal margins of eyes widely separated, by distance approximately 3x width of ocellar triangle. Gena/postocciput with fine pilosity; frons bare. Antenna large and thick; length equal to length of head, thickest portion of antenna near middle (width 0.25 × length of antenna). Flagellomeres difficult to discern, apparently 7, all but distal 2 are wider than long; flagellomere “2” [which may be 2 flagellomeres – if sulcus is present it is very obscure] twice the length of other flagellomeres; apical flagellomere small and conical. Mouthparts slightly prognathous, elements separated but difficult to discern; pair of stiff, stylate maxillae apparent, other elements probably include a labrum or hypopharynx, the labium and/or palps (segmentation of possible palps cannot be discerned).

*Thorax*: Pronotum fairly large, collar-like; mesonotum large, relatively flat; mesonotum, apical 2/3 of mesoscutellum, and anepimeron with homogeneous vestiture of fine, stiff setulae, each setula having a slightly raised, papilla-like base; row of such setulae just above wing base. Only fore leg preserved sufficiently; without spines or spurs even at apex of tibia. Empodium pulvilliform. Halter slender. *Wing*: Distal quarter lost at surface of amber. Sc complete, meeting C slightly beyond level of crossvein r-m. Vein h in line with short m-cu. Vein R_1_ straight. Vein R_2+3_ lost. Cells br and bm virtually equal in size, bisected by weak vein M. Cell m_3_ spindle-shaped, very small, approximately half the length and width of discal cell; vein M_3_+CuA_1_ incomplete (not reaching wing margin) and long, length only slightly less than length of cell m_3_. Cell cup very large, considerably thicker than and extended well beyond apical levels of cells br and bm. Vein A_1_ complete, A_2_ not apparent; alula present, but not particularly large.

*Abdomen*: Poorly preserved, genitalia lost.

**Figure 2.**
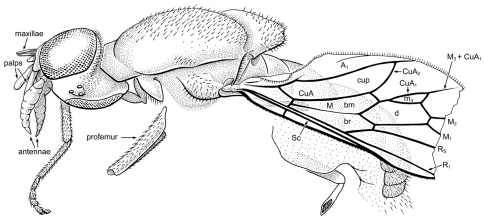
*Cretoxyla azari* Grimaldi & Cumming, gen. et sp. n. (Xylomyidae) in Early Cretaceous amber from Lebanon. Holotype, AZ391. Body length (as preserved) 3.6 mm.

##### Type.

Holotype, sex unknown, Lebanon (Early Cretaceous, Neocomian): “Hammana/Mdeiru, Aptien inférieur,” in Azar Collection no. 391, temporarily deposited in Musee National d’histoire Naturelle, Paris**.** The specimen is partially preserved, missing the right side of the body, most of the legs, and the right wing at the surface of the amber. It is mounted in a shallow glass well in Canada balsam on a glass slide.

##### Etymology.

Patronym, for Dany Azar, for his extensive contributions to the paleontology of Lebanese amber.

#### Xylomyidae (?) genus indet.

Fig. 3

##### Description.

*Head*: Lost. *Thorax*: Partially preserved, relatively broad (width of mesonotum equal to length, 1.40 mm), mesoscutellum of moderate size. Notum foveolate and scutellum covered only with numerous fine setulae, no macrosetae. Left wing 3.57 mm long, it and halter entirely preserved; right wing partially preserved. Vein C ends either at apex of R_4 _or R_5_; Sc long, meets C beyond midpoint of wing length, approximately at same level as crossvein r-m. R_1_ parallel and very close to Sc, with slightly sclerotized, pterostigmatic membrane where they diverge slightly at apex. Base of Rs (before fork of R_2+3_ and R_4+5_) short, Rs connected to R_1_ quite distad, at 0.42 complete length of wing. Veins R_4 _and R_5_ forked, branches of fork relatively straight (not curved), with R_5_ distinctively ending at apex of wing rather than below it. Cell d small, distinctively short (length 2.5x greatest width); closed cells m_3_ and cup present, cell m_3_ triangular, short branches of M_3_+CuA_1_ and A_1_+CuA_2_ present. Alula relatively small. Halter relatively short and stout. LEG: [Presumably] hind leg without macrosetae on it; presence of an empodium difficult to discern, but pulvilli well developed. *Abdomen*: Relatively broad, ending short of wing apex.

**Figure 3.**
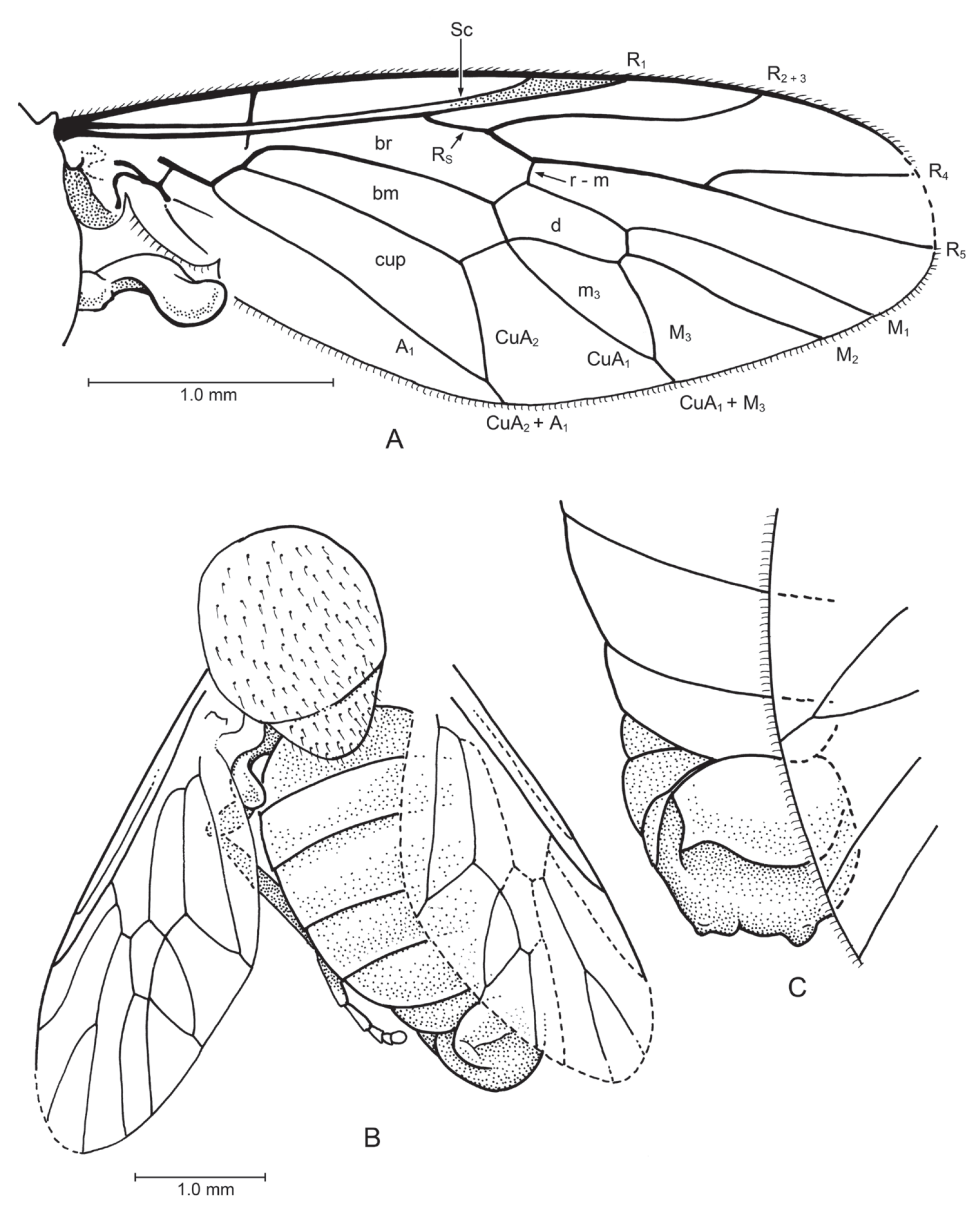
?Xylomyidae sp. in Albian amber from Spain. MCNA 8833.

##### Specimen.

MCNA 8833, Spain: Álava: Peñacerrada I, Escucha Formation, Lower Cretaceous (Albian). Specimen lacks a head, and the thorax and abdomen are only partially preserved.

##### Discussion.

Because of the incomplete preservation, a precise diagnosis and family placement of the specimen is not possible, so we did not provide a name and formal description. There are genera of lower Brachycera in several families that have a venation similar to this fossil, including the closed cell m_3_. A distinctive feature of the fossil is vein R_5_ ending at the apex of the wing. This is rarely seen in the lower Brachycera, occuring, for example, in Xylomyiidae and *Apsilocephala* Kröber, 1914 (Apsilocephalidae). Unlike *Apsilocephala*, which has the branches of R_4_ and R_5_ curved, these branches in the fossil are straight. Also, *Apsilocephala* and most therevids usually have a longer, more slender abdomen (although see *Kumaromyia*, *vide infra*), and usually have bristle-like setae on the mesonotum. These features, plus the short branch of Rs and its distal connection to R_1_ indicate that the fossil is in the Stratiomyomorpha, not the Asiloidea.

## Infraorder Tabanomorpha

### Family Tabanidae

#### Genus Cratotabanus

http://species-id.net/wiki/Cratotabanus

Cratotabanus Martins-Neto and Santos, 1994: 291. Type species: *Cratotabanus stenomyomorphus* Martins-Neto and Santos, 1994. Crato Formation (Aptian), Early Cretaceous of Brazil.

##### Diagnosis.

*Cratotabanus* isdistinguished from modern tabanids by veins M_1_, M_2_, and M_3_ long, with lengths of M_1_ approximately the same as that of cell d (vs. 0.5 – 0.7× length of cell d in Recent Tabanidae); R_5_ only slightly deviated from the path of vein R_4+5_ (in most Recent tabanids, excepting *Chrysops* Meigen 1803, R_5_ curved strongly downward). Distinguished from some Cretaceous Tabanidae, as follows: *Eotabanoid* Mostovski et al. 2003 and Yixian Formation genera with longer R_1_ (reaching well past level of apex of cell d); fork of R_4+5_ in *Eotabanoid*, *Palaepangonius* Ren, 1998, and *Eopangonius* Ren 1998 much longer (about equal in length to vein M_1_).

#### Cratotabanus newjerseyensis

Grimaldi sp. n.

urn:lsid:zoobank.org:act:B8FD8A73-669D-44EB-B923-464378B6AB94

http://species-id.net/wiki/Cratotabanus_newjerseyensis

Fig. 4

##### Diagnosis.

Venation differs from congener by *Cratotabanus stenomyomorphus* having vein R_4_ not strongly upcurved (vs. strongly upcurved) and R_5_ slightly downcurved (vs. nearly in line with R_4+5_).

##### Description.

AMNH NJ-1862 (holotype): Body length 1.0 cm, wing length 8.0 mm. Most of left lateral view and some of dorsal, right lateral, and frontal view of face observable. Specimen apparently female. *Head*: Eyes bare, large, not dichoptic, no differentiation of facets nor apparent color patterns. Details of frons and face not entirely observable (e.g., presence of frontal callus and subcallus unlikely; development of ocelli not discernable). Antenna with scape and pedicel not observable but apparently short (not projected); flagellomere I apically narrowed to 0.5 × basal width, with 3 faint annuli; remaining 6 flagellomeres stylate, tapered apicad, articles of approximately equal lengths [best seen in frontal view]. Proboscis robust, palps barely discernable (but apparently short, length 0.4 × that of proboscis), labellum well developed; entire proboscis fairly long, length = 0.75 × depth of head. *Thorax*: Standard proportions for Tabanidae; legs without discernable spurs (although apices of hind tibiae not observable). Metathoracic spiracle also not observable [e.g., presence of postspiracular scale]. *Wing*: Completely hyaline, no patterning. Base of R_2–5_ nearly perpendicular to R_1_, not at a sharp, acute angle. Fork of R_4–5_ widely divergent and encompassing entire wing tip, base of R_4_ perpendicular to R_5_, then strongly and concavely curved to meet C; base of R_4_ without a small appendix. M_1_, M_2_, M_3_ nearly parallel; M_3_ and CuA_1_ convergent (not parallel); CuA_1 _and A_1_ meeting just before wing margin. A_2 _extended nearly to wing margin; alula very large. *Abdomen*: Details (e.g., segmentation of cerci) not observable.

*Specimen.* AMNH NJ-1081 (paratype): Thorax + abdomen length 8.2 mm, wing length 8.5 mm (from base of basicosta to wing tip). Wing: Basicosta present as a thick, scale-like lobe at base of vein C. C thickened proximally, circumambient. Short crossvein h present, where costal thickening is narrowed. Sc long, 0.6 × length of wing, straight and parallel to vein C. Veins R and base of R_1_ also straight, parallel, and close to Sc; apices of Sc and R_1_ diverging apically. Dark, heavily sclerotized pterostigma covers and surrounds R_1_, vein C, and extends to tip of R_2+3_. R_2+3_ straight, turned slightly upward at apex. Stem of R_4_ and R_5_ straight, base of R_4_ nearly perpendicular to this stem, then curved upward and meeting C anterior to tip of wing; R_5_ nearly in line with stem of R_4_+R_5_. Cell d large, length ca. 2.7 × the width; with veins M_1_, M_2_ and M_3_ each deriving directly from apical wall of cell. M veins slightly divergent, long; M_1_ slightly longer than cell d, M_3_ ca. 0.6 × length of cell d. Crossveins r-m and m-cu in line with each other. Veins CuA_2_ and A_1_ meet slightly before wing margin, forming long, complete cua cell with very short vein CuA_2_+A_1_. Vein A_2_ well developed, concave to A_1_, evanescent apically; anal lobe and anal cell well developed. Alula present but partially obscure. Abdomen: Short, broad, tergites short, typical of tabanids.

**Figure 4.**
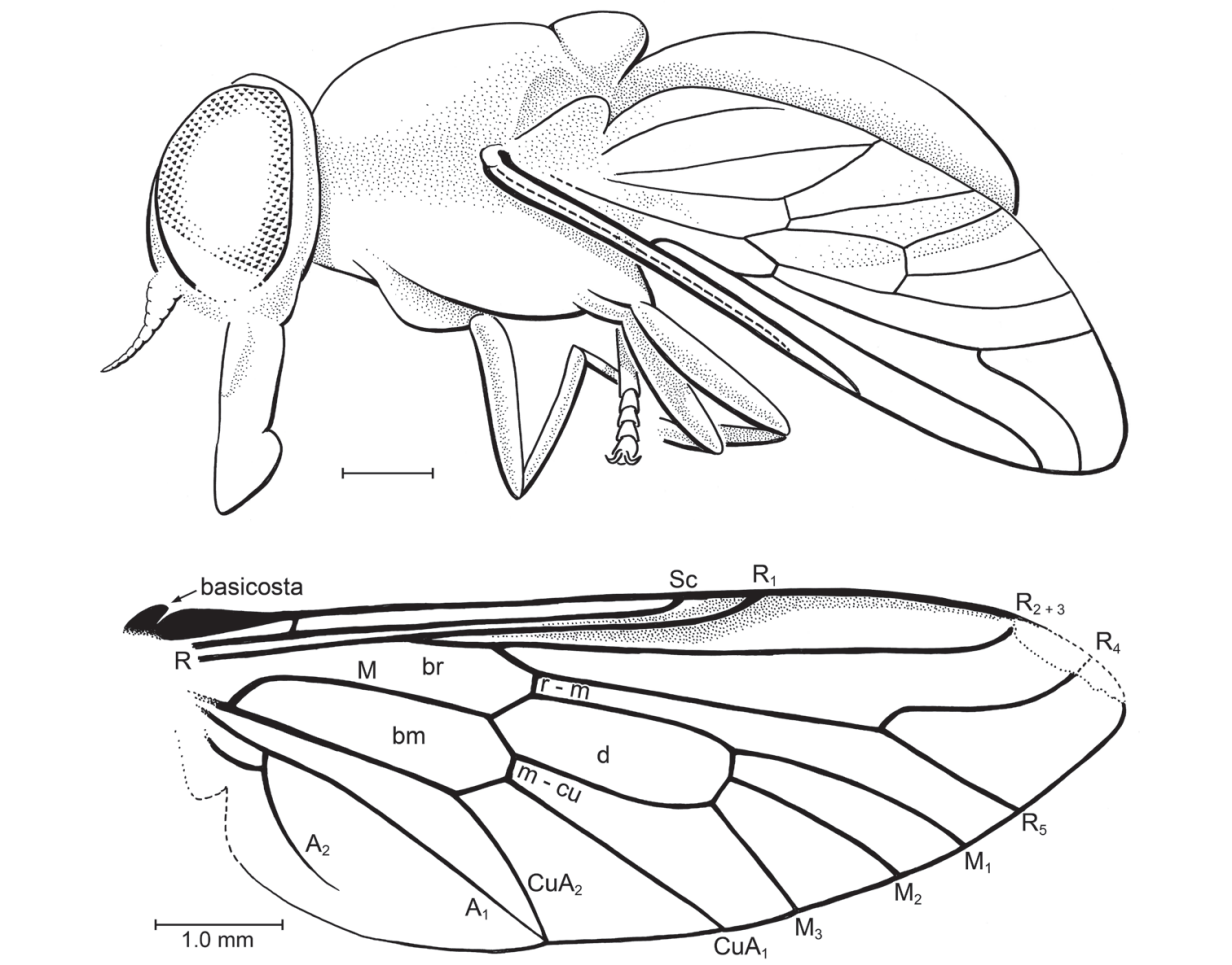
*Cratotabanus newjerseyensis* Grimaldi, sp. n. (Tabanidae) in Turonian amber from New Jersey, USA. Above: Lateral view of holotype, AMNH 1862. (scale 1.0 mm). Below: Wing of paratype, AMNH NJ1081.

##### Types.

Holotype (sex unknown), AMNH NJ-1862, New Jersey (USA): Middlesex Co., Sayreville, White Oaks [Old Crossman’s] pits (Turonian), collected by Stephen Swolensky. Observation of the fly was optimized by embedding the amber in epoxy under vacuum and trimming very close to surfaces of the fly, but the specimen is not well preserved, being occluded with a reddish, crazed layer over most of the body and by similar internal fractures in the piece, as well as by a suspension of fine particles in the amber. Piece is irregular in shape, 10 × 13 mm in largest dimensions. Study of the specimen might benefit from microtomography.

Paratype (sex unknown), AMNH NJ-1081, in Late Cretaceous (Turonian) amber from Crossman’s Pits, Sayreville, New Jersey. Fly is partially preserved: besides the entire right wing and a very small portion of left wing, only the dorsal surfaces of the abdomen and thorax remain; the head and legs are entirely lost. The amber piece is triangular and approximately 19 × 8 × 5 mm, embedded in epoxy but trimmed and polished so as to expose a dorsal view of the fly. The amber itself is light yellow and turbid, with a thick suspension of organic particles that obscures much of the fly. AMNH NJ-1081 differs from NJ-1862 by the following minor venational details: R_1_ slightly longer, Rs branches from R_1_ at a more acute angle, proximal end of cell d slightly more shallow V-shaped; A_2_ slightly shorter. Both specimens are also very similar in body shape and size.

##### Etymology.

“from New Jersey,” in reference to provenance.

##### Discussion.

These are the only tabanids known to be preserved in Cretaceous amber. Other tabanids in amber are from the Miocene of the Dominican Republic and the Eocene Baltic amber (Evenhuis 1994).

Ren (1998) described three genera of putative, compression-fossilized tabanids with long proboscides from the Early Cretaceous Yixian Formation of China. Grimaldi (1999) discussed the characters on which his assignment was made, and concluded that these fossils may not be tabanids. For example, features of *Palaepangonius eupterus* Ren that are inconsistent with Tabanidae are the short, upturned R_2+3_, very long veins R_4_ and R_5_ (half this length and much more divergent in true Tabanidae), and veins A_1_ and CuA_2_ that do not fuse but meet the wing margin independently (Fig. 4c). These do appear to be tabanomorphs, but may be stem-group taxa to Recent Tabanidae, Athericidae, Pelecorhynchidae, and possibly even Rhagionidae (some fossil rhagionids had long, piercing mouthparts). Another early compression fossil, *Baissomyia redita*, from the Early Cretaceous Zaza Formation of Russia, was attributed to the Tabanidae essentially on the basis of body shape and styletiform mouthparts (Mostovski et al. 2003), since the antennae and most of the wing (and, thus, most features defining the family) were not preserved. *Eotabanoid lordi*, from the Early Cretaceous of England, is probably a tabanid, but it too plesiomorphically has long R_4_ and R_5 _veins, which are nearly symmetrial (in true tabanids R_4_ is typically much more curved) (Mostovski et al. 2003) (Fig. 4c). Besides the specimens in New Jersey amber described herein, the only other definitive Tabanidae from the Cretaceous is *Cratotabanus stenomyomorphus* from the Aptian-aged Crato limestone of Brazil (Martins-Neto and Kucera-Santos 1994). Venation of *Cratotabanus stenomyomorphus* and *Cratotabanus newjerseyensis* are extremely similar. Another species of the genus from the Crato Formation is as yet undescribed (Martins-Neto 2003). Diverse Tabanidae occur in Tertiary rocks and amber (summarized by Evenhuis, 1994), but generic assignments of those species described prior to 1950 need to be assessed. Cretaceous fossils assigned to the Tabanidae include the following:

*Baissomyia redita* Mostovski, Jarzembowski & Coram, 2003: Zaza Formation, Baissa, Transbaikalia, Russia.

*Eotabanoid lordi* Mostovski, Jarzembowski & Coram, 2003: Durlston Formation (Berriasian), Purbeck Group, Dorset UK.

“*Allomyia*” [sensu Ren] *ruderalis* Ren, 1998: Yixian Formation, China.

*Eopangonius pletus* Ren, 1998: Yixian Formation, China.

*Palaepangonius eupterus* Ren, 1998: Yixian Formation, China.

*Cratotabanus stenomyomorphus* Martins-Neto & Santos, 1994: Crato Formation (Aptian), Ceara, Brazil.

“*Cratotabanus* sp. n.”: Crato Formation (Aptian), Ceara, Brazil (in Martins-Neto 2003: pg. 31, ex: Grimaldi 1990).

*Cratotabanus newjerseyensis* sp.n.: Raritan Formation amber (Turonian), New Jersey, USA (herein).

## Infraorder Muscomorpha

### Superfamily Nemestrinoidea Family Acroceridae

Acroceridae has been hypothesized to be closely related either to the family Nemestrinidae (Woodley 1989), or as the basal family of the Heterodactyla with the Nemestrinidae as the basal family of the Muscomorpha (Yeates 2002). Both of these families have larvae that are ectoparasitoids on other terrestrial arthropods (in the case of Acroceridae, on spiders). Relationships presented in Wiegmann et al. (2011) are extremely unusual, with Nemestrinidae close to the Tabanomorpha and Acroceridae close to Stratiomyomorpha. Nemestrinidae in particular is an ancient lineage with a distinctive wing venation, the oldest of which are Early Jurassic, and they were quite diverse by the Late Jurassic (Evenhuis 1994). An equivalent age of Acroceridae is very plausible, but Bayesian estimates of divergence between the Acroceridae and Nemestrinidae in the Triassic (Winterton et al. 2007) are almost certainly too old. The two new Burmese amber genera described below are very significant records for the family since these are the only acrocerids known from the Cretaceous, and also the best-preserved Mesozoic ones. The only other Mesozoic species of an acrocerid is from the Late Jurassic sediments of Karatau (Ussatchov 1968; Mostovski 1998), *Archocyrtus gibbosus* Ussatchev (*Juracyrtus kovalevi* Nartshuk 1996, from the same outcrops, is very similar and may even be conspecific [Hauser and Winterton 2007]). There are five acrocerid species preserved in Eocene Baltic amber. In the monophyletic subfamily Philopotinae (Winterton et al. 2007) are *Archaeterphis hennigi* Hauser and Winterton 2007 (closely related to the African genus *Africaterphis* Schlinger 1968), *Eulonchiella eocenica* Meunier 1912 (which is closely related to the Recent South African genus *Thyllis* Erichson 1840), and *Prophilopota succini* Hennig 1966. Other Baltic amber species are *Glaesoncodes completinervis* Hennig 1968 and *Villalites electrica* Hennig 1966. *Ogcodes exotica*, in Miocene Dominican amber, is closely related to several Asian species of the genus (Grimaldi 1995).

#### Schlingeromyia

Grimaldi & Hauser gen. n.

urn:lsid:zoobank.org:act: A659E121-9307-4454-B910-A586A82613E4

http://species-id.net/wiki/Schlingeromyia

##### Diagnosis.

A minute, distinctive acrocerid with medial margins of male eyes contiguous above and below antennae, hind and ventral margins of eye strongly emarginate; antennae minute, in middle of head; proboscis vestigial; eyes bare, thorax with very sparse, fine setulae; postpronotal lobes of moderate size, slightly protruding; abdomen devoid of microtrichia and glabrous (possibly reflective). Mediolobus (i.e., “pulvilliform empodium”) and pulvilli pad-like. Venation distinct: All veins sclerotized, none faint; C ends at apex of R_4+5_; Sc short; R_1_ and Rs fork at ca. 0.4× length of wing; cells br and bm continuous, not bissected (vein M extremely faint or lost from this area); two closed radial cells (r_4+5_ and d), plus cell m_3_ present; R_4+5_ ends near apex of wing, without an apical fork of R_4_-R_5_ encompassing apex of wing.

##### Type species.

*Schlingeromyia minuta*, sp. n., by present designation.

##### Etymology.

Patronym in honor of Evert Schlinger, Emeritus Professor of entomology at the University of California, Berkeley, who has devoted his career to the study of Acroceridae and who also has been a very generous patron of systematic entomology. Feminine, following the Greek *myia*, for fly.

##### Discussion.

This is a very distinctive, minute acrocerid – in body size quite the opposite of its generic namesake – which is unique for the venation, genitalia, and virtually bare body. Most acrocerids have long, fine pile on the thorax and abdomen, and many have it on the eyes and calypters. Vein Sc is very short in the fossil, and cells br and bm are contiguous. In addition, apparent retention of freely articulated gonostyli in the male genitalia appears to be a significant feature of the genus, since loss of articulated gonostyli through fusion with the gonocoxites is considered an apomorphy of the remainder of the family (Sinclair et al. 1994). The broad, pad-like structure between the pulvilli, called the empodium in homeodactylous flies, is actually a median outgrowth of the pulvilli as based on the detailed but overlooked work of Röder (1986). The true empodium is a bristle-like distal extension of the unguitractor plate. Thus, we are calling the pad-like empodium a mediolobus. Presence of a true (setiform) empodium is considered a synapomorphy of the Heterodactyla (Woodly 1989; Yeates 2002).

Winterton et al. (2007) recently analyzed acrocerid relationships based on sequences, and concluded that the subfamily Acrocerinae is diphyletic, and the subfamilies Panopinae and Philopotinae are monophyletic. Philopotines are particularly distinctive for the hump-backed notum and postpronotal lobes that are so enlarged as to form a collar dorsally over the cervical region. Although the Baltic amber *Archaeterphis hennigi* superficially resembles *Schlingeromyia* in small body size and eye shape (Hauser and Winterton 2007), the relatively complete venation of the latter, structure of male genitalia, and pretarsal structure indicate a much more basal position for the Burmese amber species. Relationships of *Schlingeromyia* to any Recent generic-group or subfamily is obscure and may reflect a stem-group position.

#### Schlingeromyia minuta

Grimaldi & Hauser sp. n.

urn:lsid:zoobank.org:act:4881BDDC-D10C-47FE-91B1-5BA12EBE2B64

http://species-id.net/wiki/Schlingeromyia_minuta

Fig. 5

##### Diagnosis.

As for the genus.

##### Description.

Body length 3.0 mm, wing length 2.1 mm. *Head*: Large, spherical. Eyes very large, occupying most of head capsule. Eyes bare, without interfacetal setulae; no dorso-ventral or frontal differentiation of facets. Entire mesal margins of eyes above antennae are contiguous, portion of mesal eye margin below antenna also contiguous. Posterior margin of eye strongly emarginate; ventral margin of eye slightly less so. Antenna minute, length approximately equal to diameter of 2–3 eye facets; consists of small oval pedicel and minute apical style. Mouthparts vestigial. Postocciput with scattered, fine setulae. *Thorax*: Scutum strongly arched, very large, length of (meso)thorax 1.25 mm (nearly half the body length). Scutellum small. Position of cervical region near ventral surface of thorax. Pair of well-developed postpronotal lobes dorsal to cervical region, posterior surface of lobe slightly concave. Scutum with sparse, short setulae; scutellum with slightly thicker setulae. Legs slender, mesotibia with short pair of apical spurs; apices of tarsomeres with pair of short, thick setae. Length of basitarsomere approximately equal to that of remaining, distal tarsomeres; hind tibia expanded in width apically to approximately twice the proximal width. Pretarsus with claws large; mediolobus and pulvilli large, pad-like. Wing short and slender, length 2.10 mm, greatest width 0.75 mm; membrane with fine, faint pleating/wrinkling over apical and posterior regions, but not in closed cells [best seen in oblique views]. Calypter large, ovoid, greatest diameter 0.58 mm. All veins sclerotized, none faint. C ends at apex of R_4+5_; Sc short, meets C slightly distal to level of where R_1_ and Rs fork. R_1_ and Rs fork at ca. 0.4× length of wing; stem of Rs short, approximately 0.2 × total length of Rs. Rs surrounds large r_4+5_ cell, near middle of which R_2+3_ branches off to meet C. Vein R_4+5_ branches off of apex of cell r_4+5_, apex meets C slightly posterior to wing apex; M_1_ also short, branching subapically off of cell r_4+5_. Tip of wing not encompassed by an apical fork of R_4_-R_5_. Cell d bounded by M_1_ and M_2_; slender (ca. 0.3 × thickness of cell r_4+5_). Cell m_3_ slender, trapezoidal, with proximal end slightly opened. Cells br and bm continuous, not bissected (vein M extremely faint or lost from this area). A_1_ slender, meeting CuA shortly before wing margin. A_2_ not apparent; anal lobe of wing well developed. Calypter large, hemispherical, greatest diameter 0.25 × length of wing. *Abdomen*: Smaller than thorax, with six tergites visible (tergite I small, tI and tII virtually obscured in dorsal view under postnotum). Tergites entirely bare of microtrichia and setulae; glabrous [probably reflective], with cuticular microsculpture of minute hexagonal cells present. Spiracles not visible near lateral margins of tergites [in pleural membrane?]. Tergites VII-VIII apparently small [not discernable]. Two pairs of male genitalic appendages present: slender dorsal pair (probably gonostyli), thicker ventral pair (gonocoxites), plus terminal, central, membranous appendage, the phallus.

**Figure 5.**
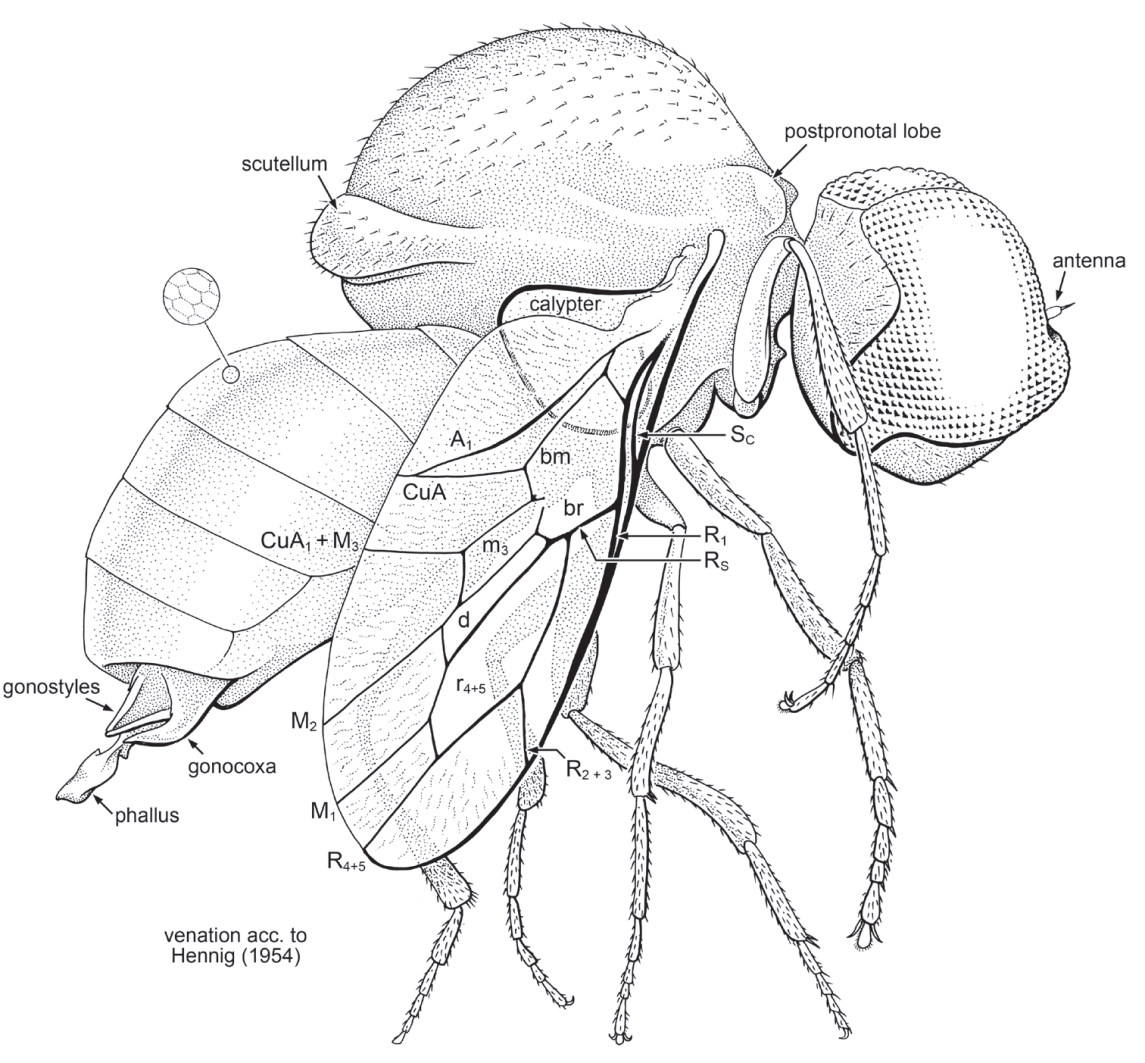
*Schlingeromyia minuta* Grimaldi & Hauser, gen. et sp. n. (Acroceridae) in latest Albian – early Cenomanian amber from Myanmar. Holotype, AMNH Bu332a. Body length 3.0 mm. Terminology for wing venation after Hennig (1954).

##### Type.

Holotype, Male, AMNH Bu332a, in Burmese amber. Paratype, AMNH Bu332b, in same piece of amber. Both specimens are entirely preserved, though slightly obscured by debris and a few small fractures. The specimens occur in a runnel-shaped piece of dark but transparent amber, 16 × 7 mm, which has been embedded in epoxy. The piece also contains 1 Coleoptera, 1 Hymenoptera (Serpitidae), and 6 other Diptera (Cecidomyiidae, Empidoidea), as well as twisted strands of spider webs. Interestingly, acrocerids are parasitoids of spiders.

##### Etymology.

Latin, adjective, in reference to the very small size of the species.

#### Burmacyrtus

Grimaldi & Hauser gen. n.

urn:lsid:zoobank.org:act:622EFA4E-5D9E-4244-8213-D20654E1AE15

http://species-id.net/wiki/Burmacyrtus

##### Diagnosis.

A small, primitive acrocerid in Burmese amber easily separated from *Schlingeromyia* based on the well developed mouthparts; long, fine antennal stylus; dense, fine pilosity on thorax and abdominal tergites; absence of a mediolobus on the pretarsus; absence of tibial spurs; wing apex rounded; and by the venation: Vein C circumambient, cells br and bm completely separated, absence of cells r_4+5_ and m_3_, presence of a very large cell d, vein R_4_ present but vestigial (not connected to R_5_), veins CuA_1_ and CuA_2_ each present, vein A_1_ vestigial (cell cup not present).

##### Type Species.

*Burmacyrtus rusmithi* sp. n., by present designation.

##### Etymology.

Combination derived from Burma (the pre-junta name for Myanmar) and *Cyrtus*, nominal genus of Cyrtidae, a formerly used name of Acroceridae.

##### Discussion.

Derived acrocerid features that *Burmacyrtus* shares with *Schlingeromyia* and Recent acrocerids are the following: spherical head with large, holoptic eyes in male; apex of antennal flagellum with simple stylus; presence of a distinct cervical region; wing membrane with fine wrinkling and devoid of microtrichia; and with a large calypter. Apomorphic features in *Burmacyrtus* that are lacking in *Schlingeromyia* are fine, dense pilosity; a broadly rounded wing apex; long, fine stylus; and lack (loss) of a mediolobus. The wing shape of *Burmacyrtus* is similar to that of some Recent acrocerine genera such as *Turbopsebius* Schlinger, 1972, but the latter genus has cell r_4+5_ present, veins CuA_1_+M_3_ fused, and a complete vein A_1_, among other features. Like *Schlingeromyia*, *Burmacyrtus* is also very basal in the Acroceridae. Some of the derived features in wing venation of the two species in Burmese amber may be due to the very small body size.

#### Burmacyrtus rusmithi

Grimaldi & Hauser sp. n.

urn:lsid:zoobank.org:act:17FA87A4-457F-4BB0-AC59-03ECEBF4CAA4

http://species-id.net/wiki/Burmacyrtus_rusmithi

Fig. 6

##### Diagnosis.

As for genus.

##### Description.

Wing length approximately 1.4 mm, body length approximately 2.0 mm. *Head*: Rounded, spherical. Male eyes bare of setulae, frontally holoptic [dorsum of head not visible], occupying most of head capsule, ventromesal margins of eyes diverging around clypeus; facets in ventral portion of eye not differentiated in size; posteroventral margin of eye with shallow emargination. Basal portion of flagellum small, ovoid; stylus long and very slender, length c. 2 × that of basal portion; apex of stylus with pair of minute setulae. Labellum well developed; palps not evident. Postocciput with dense, fine pilosity. *Thorax*: Cervical region elongate, but not comprised of elongate postpronotal lobe (which protrudes slightly from anterior surface of scutum); cervical region connected anteroventrally to thorax. Thorax deep. Dorsal surface of mesoscutum and scutellum with dense, fine pilosity. Legs slender, metafemur longest; without spines, bristle, or tibial spurs; tibial and tarsal setulae not in regular rows. Apical portions of tibiae not distinctly broadened. Pretarsal claws large; pulvilli large, mediolobus absent [if setiform empodium present, not visible]. Wing short, with broadly rounded apex and narrow base; surface devoid of microtrichia, with fine wrinkling throughout. Vein C circumambient, though thinner past apex of M_1_; small hump in C midway along length of Sc. Sc complete, length ca. 0.4 × that of wing (a thin, faint, incomplete, and apparently spurious vein runs parallel and very close to Sc). Vein R_1_ short, length approximately 0.5 × length of stem of R; R_1_ and C thickened where they meet. Stem of Rs short, length approximately 0.5 × that of R_1_; Rs and where it meets M thickened. R_4_-R_5_ apparently a vestigial fork (R_4 _incomplete, not connected to R_5_). Cells r_4+5_ and m_3_ absent; cell d present, large; length of cell d 0.3 × that of wing. Cells br and bm present, separated by well developed basal portion of M. Veins M_1_, M_2_, M_3_ present, originating from apex of cell d. Veins CuA_1_ and CuA_2_ present, originating from apex of cell bm. Vein A present, but short and vestigial (cell cup absent). Alula and calypter well developed, each with fine wrinkling; calypter approximately 2 × diameter of alula. Halter apparently dark. *Abdomen*: larger than thorax; sternites well developed, glabrous, without setulae or punctures. Tergites large, with dense, file pilosity; each setula situated in minute puncture. Male genitalia: epandrium well developed, shallow; cerci slender and apically pointed; everted, distal portion of phallus bulbous; subapical portion flanked by pair of flat, setulose lobes. Spiracles not visible.

**Figure 6.**
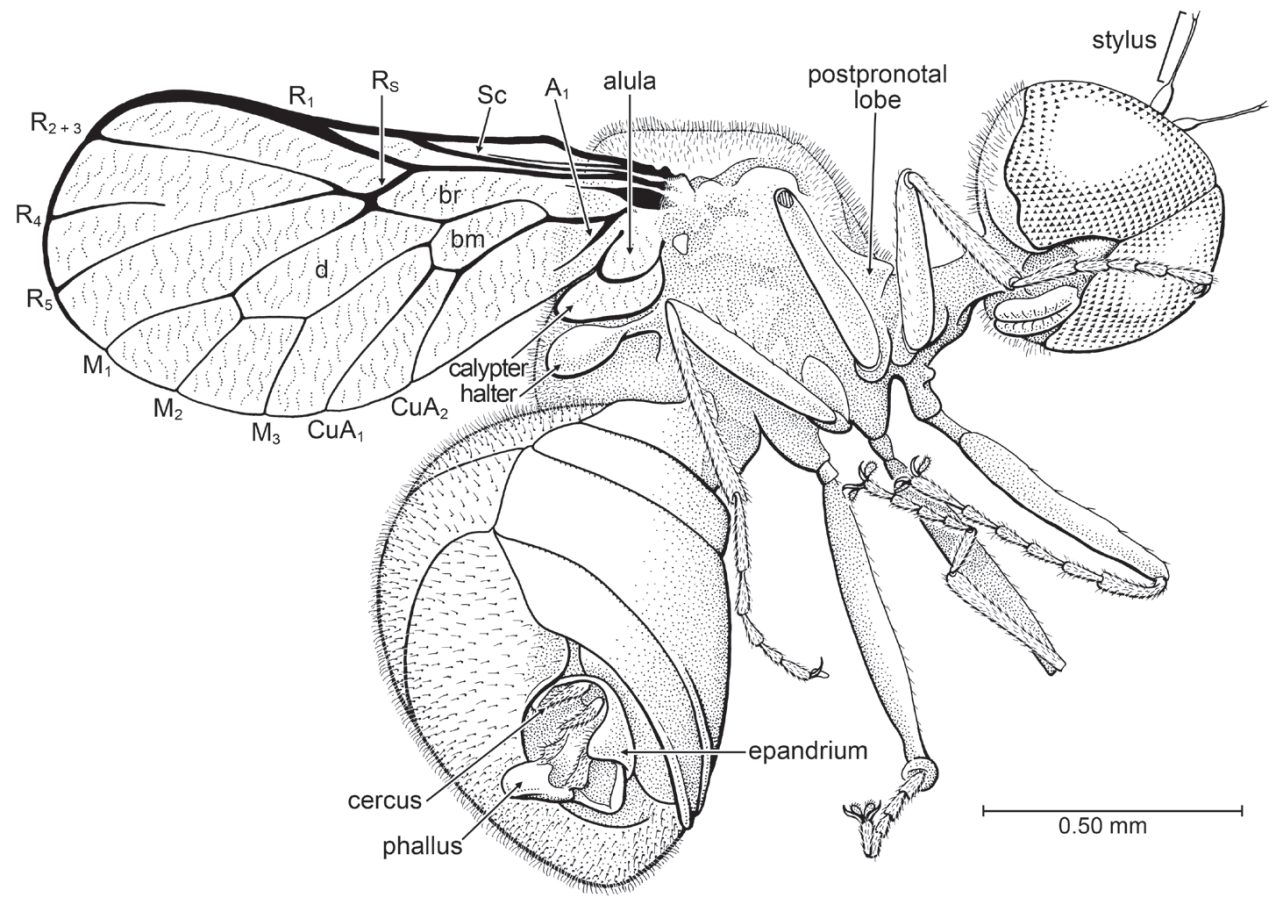
*Burmacyrtus rusmithi* Grimaldi & Hauser, gen. et sp. n. (Acroceridae) in Burmese amber. Holotype AMNH Bu-RS1.

##### Type.

Holotype, Male, AMNH Bu-RS1, in Burmese amber. The holotype is in excellent condition, though only the ventral and lateral portions are visible (the dorsal surface is obscured by the depth and curvature of the amber). The amber is clear yellow and the fly lies on an internal surface plane that contains bubbles and stellate trichomes. The original piece was drop-shaped, 10 × 16 mm, and contained a small spider, cecidomyiid midge, and berothid lacewing. These inclusions were separated from the fly.

##### Etymology.

Patronym, for Dr. R.D.A. (Ru) Smith, who generously donated the specimen to the AMNH from his personal collection.

## Superfamily Asiloidea **Family Mythicomyiidae**

### Microburmyia

Grimaldi & Cumming gen. n.

urn:lsid:zoobank.org:act:7C83B2CC-BD8F-4BDA-A19D-E61110619770

http://species-id.net/wiki/Microburmyia

#### Diagnosis.

R_1_ long, apex reaching to 2/3 length of wing; R_1_ branching off of the stem of R quite distad, R_2+3_ long, branching off of Rs in the distal half of the wing; cells br and bm large, length nearly half that of wing; M_1+2_ forked; vein A_1_ either incomplete or absent. Mesoscutum strongly arched; it and abdominal tergites devoid of bristle-like setae or long pilosity; apical tibial spurs lacking. Body size minute, ca. 1.0 mm in length.

#### Type Species.

*Microburmyia analvena*, sp. n. By present designation

#### Etymology.

Derived from *micro*- (L.), minute; -*burm*-, Burma; and –*myia* (Gr.), fly, in reference to the minute body size and provenance of this brachyceran. Feminine.

#### Discussion.

The family placement of the two new species in this genus is not entirely certain, particularly since in mythicomyiids vein R_2+3_ is typically short and its apex fused with R_1_. The genus is placed in the Mythicomyiidae since the venation bears a resemblance to the Baltic amber genus *Carmenelectra* Evenhuis (Evenhuis 2002), though differing from that Baltic amber genus by R_1_ branching off of the stem of R quite distad (vs. near the base of the wing), R_2+3_ branching off of Rs in the distal half of the wing (vs. in the basal half), and by crossveins r-m and cu-m being in line (vs. stepped). Other features of *Microburmyia* that are consistent with mythicomyiids are the strongly arched scutum, structure of apical antennal articles (with minute stylus in *Microburmyia analvena*, ovoid first flagellomere in *Microburmyia veanalvena*), incomplete Sc vein, palpi minute or absent, and the minute body size. *Microburmyia* is plesiomorphic with respect to all other known mythicomyiids (cf., Evenhuis 2002).

Mythicomyiidae are traditionally (e.g., Hall 1981) and phylogenetically (e.g., Yeates 1994; Woodley 1989; Woodley et al. 2009) placed as the sister group to the Bombyliidae s.s, often classified as a subfamily, but also as a separate family (e.g., Evenhuis 2002). There is strong morphological and molecular support for Bombyliidae + Mythicomyiidae being the sister group to the rest of Asiloidea (Woodley 1989; Yeates 2002; Wiegmann et al. 2011) or the sister group to the rest of Asiloidea and Eremoneura (Sinclair et al. 1994; Trautwein et al. 2010). With the exception of an unforked R_4+5_ and short or vestigial anal vein in *Microburmyia*, it is very interesting that its venation is intermediate between that of the Hilarimorphidae and the more specialized venation of mythicomyiines. This would lend support to the hypothesis that Hilarimorphidae is the sister group to the Bombyliidae (Woodley 1989; Yeates 1994; Woodley et al. 2009). Another hypothesis places the Hilarimorphidae (sometimes including the enigmatic and monotypic genus *Apystomyia* Melander 1950) as the sister group to the Eremoneura (Yeates, 2002) (see also discussion under Apystomyidae, *vide infra*). Oddly, the recent total-evidence phylogeny of flies placed the Hilarimorphidae (excluding *Apystomyia*) as sister group to the Acroceridae (Wiegmann et al. 2011), for which there is very limited molecular and no morphological support, although this result does suggest a position of Hilarimorphidae distant from Eremoneura.

Lastly, it is interesting to note that the fossil record of Bombyliidae s.s., exclusive of mythicomyiines, is entirely Tertiary. Bombyliidae is a large, cosmopolitan family (ca. 4,500 species) of flies that are most diverse in xeric ecosystems, where they are important pollinators of herbaceous plants. Their fossil record in sedimentary matrices and in amber (Baltic, Dominican) is quite diverse for North America and Europe (Evenhuis 1994), suggesting that the bombyliids s.s. radiated rapidly in the early Tertiary.

### Microburmyia analvena

Grimaldi & Cumming sp. n.

urn:lsid:zoobank.org:act:E64C24A4-F610-4034-AC24-D65721D5BEF5

http://species-id.net/wiki/Microburmyia_analvena

Fig. 7a, b

#### Diagnosis.

Distinguished from *Microburmyia veanalvena* sp. n. (below) by longer wing; presence of an anal vein; fringe of fine (vs. thick) setae on posterior wing margin; antennal style very fine, with very small article between it and basal flagellomere.

#### Description.

A minute fly, body length c. 1.1 mm, thorax length 0.5 mm, wing length 1.15 mm. *Head*: Short, somewhat flattened anteroposteriad. Cervical region long. Eyes bare, large, well separated; no dorsoventral differentiation of facets; with small, shallow emargination on posterior margin. Proboscis short [palps not visible]. Antenna with basal flagellomere drop-shaped, with sparse setulae; apical style 0.6 × length of basal flagellomere, very thin, with two articles. Three ocelli present. Postocciput expansive, concave. *Thorax*: Mesoscutum dorsally arched, devoid of setae or setulae; thorax deep in lateral view; mesoscutellum triangular in shape (nearly equilateral), posterior end tilted upward. Coxae of moderate size; legs slender; devoid of setae, tibiae without apical spurs. Pretarsus with large pulvilli; empodium probably setiform. Halter with slender stem, large knob. Wing long, length slightly greater than length of body, wing L/W = 2.72. Costa either without spinules or spinules minute; C reaching slightly beyond apex of R_4+5_. Posterior margin of wing with fringe of short, fine setae, including alula (setae longer in this area). Vein Sc with apex apparently evanescent, not reaching C. R-R_1_ nearly straight; R_2+3_ 2.0 × length of R_1_; R_4+5_ straight, ends at apex of wing; proximal portion of R_4+5_ joined to r-m to form distal margin of cell br. Cells br and bm large, br is 0.33x length of wing, W/L cell br = 0.3; cell bm narrower and shorter. Base of M straight, with short apical fork. Crossvein bm-cu slightly shorter than r-m, not in line with each other. CuA_1_ and CuA_2_ short, curved slightly toward each other. Vein A_1_ present, incomplete (reaching to 0.6x distance between vein base and wing margin), apex of vein blunt, not evanescent. Anal lobe and alula small. *Abdomen*: Short, 1.3 × length of mesothorax, apparently devoid of setae and setulae. Tergites I – V with shallow, median keel; epandrium with pair of large ventral lobes.

**Figure 7.**
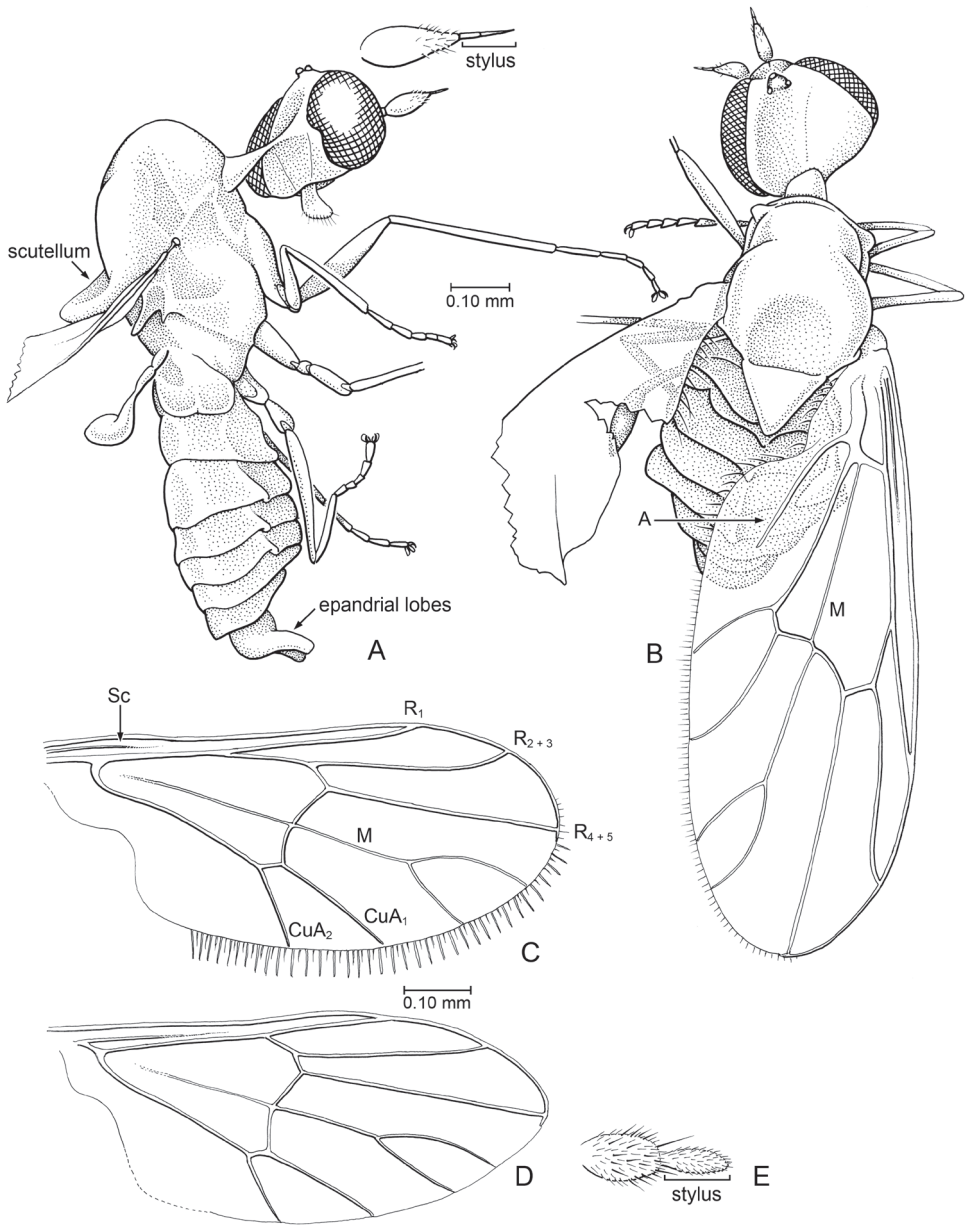
*Microburmyia* Grimaldi & Cumming, gen. n. (Bombyliidae: Mythicomyiinae), in Burmese amber **a, b** *Microburmyia analvena* Grimaldi and Cumming sp. n. Holotype, KU-Bu079 (**a** lateral view, with detail of antenna **b** dorsal view, as preserved) **c – e** *Microburmyia venanalvena* Grimaldi and Cumming, sp. n., Holotype AMNH Bu1552 **c, d** left and right wings, showing variation in vein proportions. e, antenna.

#### Type.

Holotype, male: Myanmar: Kachin (northern Myanmar), in Burmese amber, KU Bu079 (Univ. Kansas, Division of Entomology, Natural History Museum). The amber piece containing the holotype is a very transparent, deep amber color, 14 × 7 × 5 mm, which also contains 2 scelionid wasps. The minute holotype is at the surface of a fractured corner.

#### Etymology.

in reference to the presence of an anal vein (i.e., the Latin noun *vena*), albeit incomplete.

### Microburmyia veanalvena

Grimaldi & Cumming sp. n.

urn:lsid:zoobank.org:act:440657CB-BAD8-4D35-8F8A-1D03B5752FB0

http://species-id.net/wiki/Microburmyia_veanalvena

Fig. 7c-e

#### Diagnosis.

cf. *Microburmyia analvena* (above), distinguished by the absence of an anal vein; posterior fringe of setae long, thick; basal flagellomere and style setulose, style with one article, oval.

#### Description.

A minute fly, wing length 0.85 mm. *Head*: Short, somewhat flattened anteroposteriad. Cervical region with connection anteroventrally on thorax; not visible dorsally. Eyes bare, large, well separated; no dorsoventral differentiation of facets [presence of emargination on posterior margin not visible]. Proboscis short [palps not visible]. Antenna with basal flagellomere ovoid, having dense setulae (longer apicad); apical style 0.6 × length of basal flagellomere, thick (nearly 0.5 × thickness of basal flagellomere), one articled, setulose. Three ocelli present. Postocciput expansive, concave. *Thorax*: Mesoscutum dorsally arched, devoid of setae or setulae; thorax deep in lateral view; mesoscutellum triangular in shape (nearly equilateral). Legs slender; devoid of setae, tibiae without apical spurs. Pretarsus with large pulvilli; empodium probably setiform. Halter with slender stem, large knob. Wing W/L = 0.43. Costa either without spinules or spinules minute; C reaching slightly beyond apex of R_4+5_. Posterior margin of wing with fringe of long, thick setae (visible only on left wing), but margin of alula bare. Vein Sc extremely faint, evanescent. R-R_1_ nearly straight; R_2+3_ 2.0 × length of R_1_; R_4+5_ straight, ends at apex of wing; proximal portion of R_4+5_ joined to r-m to form distal margin of cell br. Cells br and bm large, br is 0.42 × length of wing, W/L cell br = 0.27; cell bm slightly narrower and shorter. Base of M straight, with short apical fork. Length crossvein bm-cu approximately equal to that of r-m, in line with each other. CuA_1_ and CuA_2_ short, straight and diverging. Vein A_1_ absent. Anal lobe differentiated, alula small. *Abdomen*: Very broad anteriorly, devoid of setae and setulae on tergites. Tergites I – V apparently without shallow, median keel [difficult to discern with preservation]; epandrium with 5–6 long, thick setae on posterior surface, length of setae approximately equal to length of epandrium.

#### Type.

Holotype, Male: Myanmar: Kachin (northern Myanmar), latest Albian to earliest Cenomanian. AMNH Bu1552. Specimen is displayed with wings and legs outspread, but body is only moderately well preserved, with some details obscured beneath layer of deep reddishness. Dorsal view is better than ventral view.

#### Etymology.

*ve*- (Latin prefix meaning without), anal vein (L., *vena*), in reference to this venational character.

#### Discussion.

It could be argued that these two species might warrant separate genera, based on the differences of antennae, wing fringe, epandrial setae, and proportions of the wing. However, other than the presence/absence of the anal vein, the wing venation is very similar between the two species.

## Therevidae family group

This asiloid group includes the Recent families Therevidae (cosmopolitan; 1,063 described species), the Scenopinidae (cosmopolitan, approximately 420 described species), the monotypic family Evocoidae from Chile (Yeates et al. 2003, 2006), and the small, relict family Apsilocephalidae. Species in the group are most diverse in and largely adapted to dry habitats. Apsilocephalidae is comprised of *Apsilocephala* Kröber (one extant and several undescribed ones species from western North America, plus two fossil species [see below]), *Burmapsilocephala* Gaimari & Mostovski, 2000 (one species in Burmese amber), *Clesthentia* White (two species, from Tasmania), and *Kaurimyia* Winterton and Irwin (one species, from New Zealand) (Yeates et al. 2003; Winterton and Irwin 2008). Monophyly of the family group is not disputed, though hypotheses of relationships among the four families differ slightly (Yeates 2002; Yeates et al. 2003; Wiegmann et al. 2011), and the possibility exists that Therevidae is paraphyletic with respect to Scenopinidae (Woodley 1989; Yeates et al. 2007).

### Family Apsilocephalidae, or near

#### Kumaromyia

Grimaldi & Hauser gen. n.

urn:lsid:zoobank.org:act:08C3574B-22B5-4654-99CE-3DE0488EDBA2

http://species-id.net/wiki/Kumaromyia

##### Diagnosis.

Body stout, abdomen short (length about equal to that of thorax); eyes large, bare; antenna with 3 flagellomeres, second article and third (style) minute; palp one-segmented; legs and thorax with bristle-like setae, no pilosity except for postoccipital region; hind coxa with small knob on anterior surface; thickness of metatarsi equal that of metatibial base; wing with C ending between apices of R_5_ and M_1_, apex of R_5_ ending slightly subapically; R_4_ and R_5_ divergent, not parallel for any part of their lengths, base of R_4_ not perpendicular to stem of R_4+5_ and R_5_ .

##### Etymology.

Patronym in honor of a great colleague and friend to the senior author, Prof. Kumar Krishna. Appropriately, *Kumaromyia* (as presently known) is preserved in amber from Burma, a place of significance in Kumar’s early years.

##### Type species.

*Kumaromyia burmitica*, sp. n., by present designation.

##### Discussion.

*Psilocephala electrella* Cockerell, 1920, is a similar species preserved in Burmese amber, but *Kumaromyia burmitica* has a smaller body size (wing width 0.75 mm, vs. 1.5 mm in holotype of *electrella*), and differs venationally, specifically with the apex of R_5_ meeting C preapically (vs. slightly postapically in *electrella*), M_1_ and M_2_ nearly parallel (distinctly divergent in *electrella*), and apex of M_3_ distinctly curved to meet apex of CuA_1_ at the wing margin (vs. straight in *electrella*). The holotype and unique specimen of *electrella* (NHML In. 20148) is shown in an excellent photograph in Gaimari and Mostovski (2000: fig. 1), and it was examined by the senior author in 2004. It is very partial, with most of the specimen lost (just portions of the scutum and abdomen, two legs and most of both wings remain), so it is very difficult to determine if the two species may be congeneric.

#### Kumaromyia burmitica

Grimaldi & Hauser sp. n.

urn:lsid:zoobank.org:act:96FE6FDB-D380-47A8-8EDB-2FE979761B72

http://species-id.net/wiki/Kumaromyia_burmitica

Fig. 8

##### Diagnosis.

As for the genus.

##### Description.

Small fly, total body length ca. 2.70 mm, thorax length 1.0 mm, wing length (estimated) 2.50 mm. *Head*: Large, with large eyes. Eyes bare, hemispherical in lateral view (posterior margin flat), no dorsoventral differentiation of facets; inner margins of eyes parallel, separated by distance approximately equal to width between antennal bases. Frons slightly convex, not protruding anteriad; with numerous fine setulae, without calli. Face (“subcranial cavity”) depressed, dark (sclerotized?), glabrous. Antennal scape and pedicel small, approximately equal in size, devoid of thick setae; basal flagellomere largest antennomere, drop-shaped, with dense setulae (no setae); apical two antennomeres (including apical style) small, fine, with style slightly longer than penultimate antennal article. Maxilla with bases (cardostipites) sclerotized and partially fused, palp1-segmented. Labellum slightly larger than palps. Postgena well developed, with numerous fine setae (pilosity). *Thorax*: Deep in lateral view, pleura apparently devoid of fine or bristle-like setae; scutum with at least 8 pairs of setae [dorsal view, including scutellum, obscured]. Scutum with 3 pairs of notopleurals and 5 pairs in supra-alar region and some setulae; no cervical/postcervical setae. *Legs*: With thick, stiff setae, primarily on tibiae; fore tibia slender, hind tibia thickest. Fore leg: femur with lateral row of ca. 10 fine setae, tibia with anterior row of 4–5 setae, 4 pre-apical setae. Mid leg: Femur apparently devoid of setae, tibia with 3 evenly-spaced setae on dorsal surface, 2 more ventrad, 4 apically. Hind leg: Coxa with small knob on ventral surface [best seen in left coxa]; femur devoid of setae, tibia with dorsal row of 3–4 setae, lateral row of 3 setae, ventral row of 3–4 setae. Basitarsomere on each leg equal in length to (or slightly longer than) combined length of distal tarsomeres. Each tarsomere with ca. 4 short, stiff setae on rim of distal end. Pretarsus with pair of large pulvilli, empodium setiform. *Wing*: Large, length nearly equal to that of body. Crossvein h long (space between Sc and C deep); Sc long, length approximately ½ that of wing and slightly shorter than length of R1; apex of Sc apparently incomplete (not meeting C). Apices of Sc and R_1_ without pterostigma surrounding apices. Fork of R and Rs deep, proximal to level of vein h. R_2+3_ straight, without apical curve. Fork of R_4+5_ not widely divergent; R_5_ in line with stem of R_4+5_, apex of R_5 _ending very near apex of wing (not posterior to it); R_4_ slightly curved, distinctly shorter than R_5_. Cell d slender, greatest width <0.25 × length. Veins M_1_ and M_2_ slightly divergent, M_2_ and M_3_ very divergent, all M veins attached to apex of cell d. Apex of M_3_ meeting apex of CuA_1_ at wing margin. ABDOMEN: Short, only slightly longer than thorax; details (e.g., sternites, genitalia) not observable.

**Figure 8.**
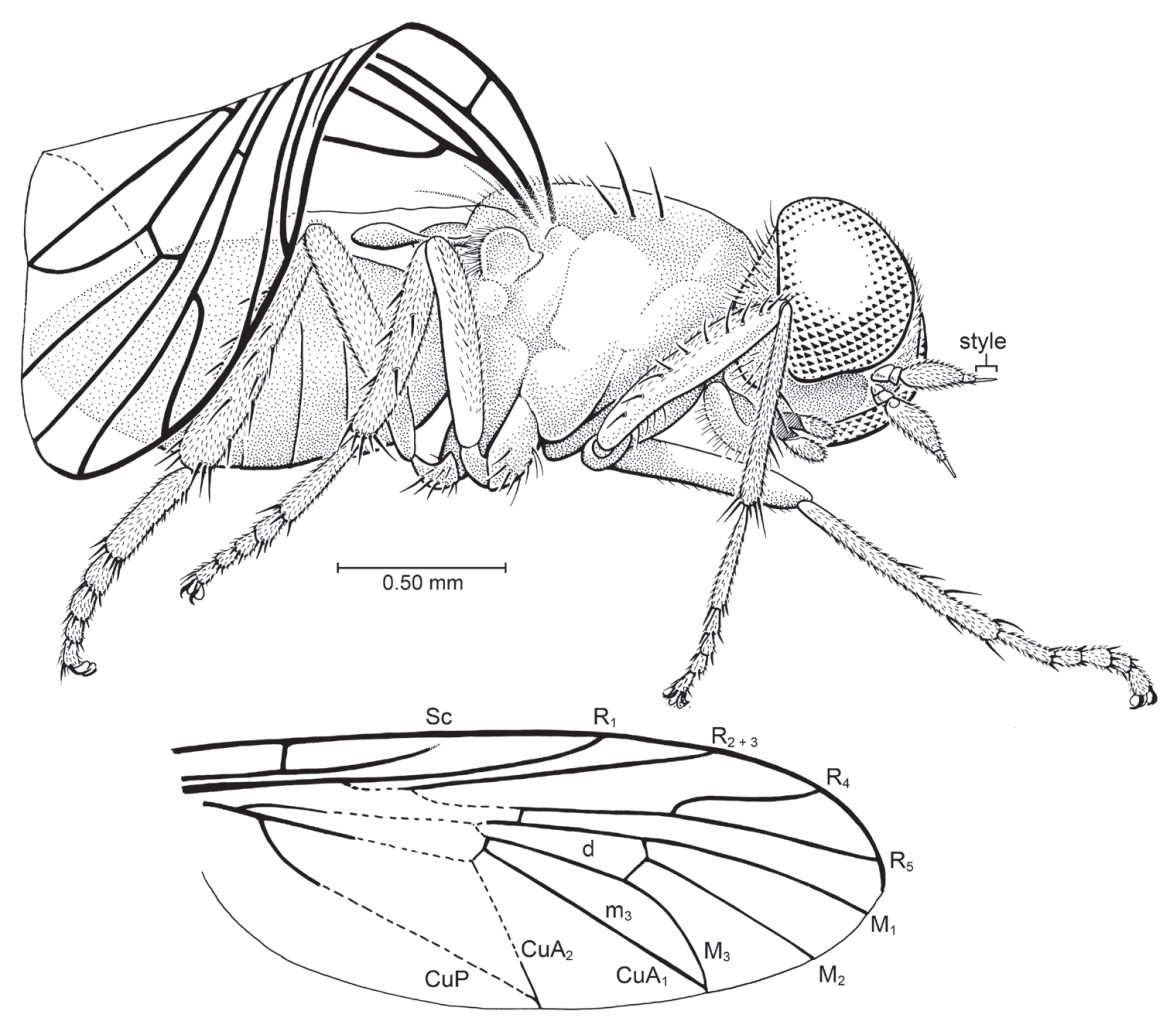
*Kumaromyia burmitica* Grimaldi & Hauser, gen. et sp. n. (Therevoid family group: ?Apsilocephalidae), in Burmese amber. Right lateral habitus of holotype AMNH Bu131, as preserved. Below: wing, partially reconstructed.

##### Type.

Holotype, female, AMNH Bu131: Myanmar: Kachin State, near Mytikyina (mid-Cretaceous: Late Albian – Cenomanian). Specimen is complete, but the right wing (the only one observable) is folded, and most of the dorsal view is obscured, compromising a complete reconstruction of the venation (fig. 8). The fly is complete, though slightly compressed and with a slight coating of particulate matter over some areas. Its left side is lying on a rough surface of the amber, which obscures that view. The piece also contains some twisted strands of spider webbing.

##### Etymology.

In reference to the country of origin.

##### Discussion.

There is little question this fossil belongs to the therevid group, albeit unusually small (within the range in body size of some apsilocephalids and a few genera of Phycinae, such as *Efflatouniella* Kröber, 1927). Therevid-group features include the antennal structure, bristle-like setae on the scutum and on the legs, the small knob on the hind coxa, as well as the venation. Unlike most Therevidae, *Kumaromyia* lacks any pruinosity and pilosity (except for the postgena), although Xestomyzinae and Agaphotinae are also robust and have sparse pilosity. *Kumaromyia* lacks any thick setae that typically encircle the scape and/or pedicel subapically in Therevidae. Also, *Kumaromyia* has R_4_ and R_5_ above the wing tip, whereas in Therevidae these are above and below the wing tip, respectively. Unlike Apsilocephalidae, *Kumaromyia* has a one-segmented palp, vs. two-segmented in Apsilocephalidae, where the basal segment is distinctively thin and long (oddly, palp segmentation and structure was not described for *Kaurimyia*). The antennal stylus and stout body in *Kumaromyia* is much more similar to that of *Clesthentia*, as the stylus in *Apsilocephala*, *Kaurimyia*, and even *Burmapsilocephala* is long and thin. It is quite possible that *Kumaromyia* is a stem-group taxon for the therevid-family group, not necessarily belonging within Apsilocephalidae or Therevidae.

Fossil Therevidae are scarce, with only five definitive species known, all from the Tertiary. Hauser (2007) and Hauser and Irwin (2005) revised the fossil species:

*Ambradolon grimaldii* Metz and Irwin 2000: Early Miocene Dominican Republic amber

*Arctogephyra agilis* (Meunier 1908): mid-Eocene Baltic amber

*Dasystethos hoffeinsi* Hauser 2007: mid-Eocene Baltic amber

*Kroeberiella pinguis* (Loew 1850): mid-Eocene Baltic amber

*Palaeopherocera scudderi* (Cockerell 1909): uppermost Eocene, Florissant, Colorado, USA

Fossil Apsilocephalidae range from the Cretaceous to early Tertiary:

*Apsilocephala pusilla* (Hennig 1967): mid-Eocene Baltic amber

*Apsilocephala vagabunda* (Cockerell 1927): uppermost Eocene, Florissant, Colorado, USA

*Burmapsilocephala cockerelli* Gaimari and Mostovski 2000: mid-Cretaceous Burmese amber

Undescribed sp.: Early Cretaceous amber, Wealden, UK (Chandler 2010: plate 32, fig. 2).

The position of *Psilocephala electrella* Cockerell 1920 within the therevoid group is uncertain.

## Families Incertae sedis

### Family Apystomyiidae

This family contains the sole Recent species *Apystomyia elinguis* Melander, 1950, from California, one of the world’s most relict and intriguing flies, with a dramatic history of systematic interpretation. Traditionally, *Apystomyia* has been placed in the Bombyliidae (e.g., Hall 1981), and then was hypothesized to be the sister group to the Eremoneura (Wiegmann et al. 1993). It has been placed in the Hilarimorphidae (Yeates 1994), as well as allied to the Therevidae (Sinclair et al. 1994). Nagatomi and Liu (1994) concluded that the male and female terminalia differ markedly from Hilarimorphidae, with the female terminalia similar to those of the Cyclorrhapha, and they erected the Apystomyiidae for this species. Soon thereafter, Nagatomi (1996) indicated that *Apystomyia* is allied to the proratine Scenopinidae. More recently, Yeates (2002), in his morphological analysis of basal Brachycera relationships, hypothesized the Hilarimorphidae (including *Apystomyia*) as being the sister group to the Eremoneura. However, Trautwein et al. (2010) considered *Apystomyia* to be the sister group to the Cyclorrhapha based on molecular evidence. Lastly, the comprehensive total-evidence study by Wiegmann et al. (2011) proposed a sister-group relationship of *Apystomyia* to the Cyclorrhapha, separate from Hilarimorphidae (which was placed near Bombyliidae).

### Genus Hilarimorphites

*Hilarimorphites* Grimaldi & Cumming, 1999: 21. Type species: *Hilarimorphites yeatesi* Grimaldi and Cumming. By original designation.

#### Hilarimorphites burmanica

Grimaldi & Cumming sp. n.

urn:lsid:zoobank.org:act:BAEACD0A-8879-4761-95DB-4AF8297CADA0

http://species-id.net/wiki/Hilarimorphites_burmanica

Fig. 9

##### Diagnosis.

Distinguished from the 4 other species in the genus (known only in New Jersey amber) by venation: vein C ending just slightly beyond apex of R_4_ (not at apex of R_5_); Sc long, distally incomplete (more so than in *Hilarimorphites superba* Grimaldi and Cumming 1999, the only other species with this trait); veins CuA_2_ and A_1_ not joined before meeting wing margin (anal cell open distally). Distinguished from *Apystomimus* by the larger (normal-sized) wings, with an open cup (anal) cell. Also, basal flagellomere is more elongate and triangular in *Hilarimorphites burmanica*, and the antennal stylus longer than in the other species of *Hilarimorphites*.

##### Description.

Based on a virtually complete, well-preserved female. Body length (excluding antennae) 1.40 mm; thorax length 0.50 mm; wing length 0.95 mm. *Head*: Antenna with first flagellomere an elongate triangle in lateral view; apical antennal article(s) form a thin style, with possibly a minute apical article. Eyes large, glabrous. Frons with sparse, scattered setae. Proboscis with broad, flat labellum (palps not visible). *Thorax*: Notum dome-shaped, with sparse, fine, stiff setae; scutellum with 2 pairs of erect setae. Legs very slender, of moderate length, without distinctive spines or tibial spurs. *Wing*: typical of *Hilarimorphites*, except as given in diagnosis above [also, anal lobe may be less developed than in other species, but this area slightly folded under and obscured]. Halter of moderate length, knob slender. *Abdomen*: Slender, tergites unmodified, cerci and genitalia not fully visible.

**Figure 9.**
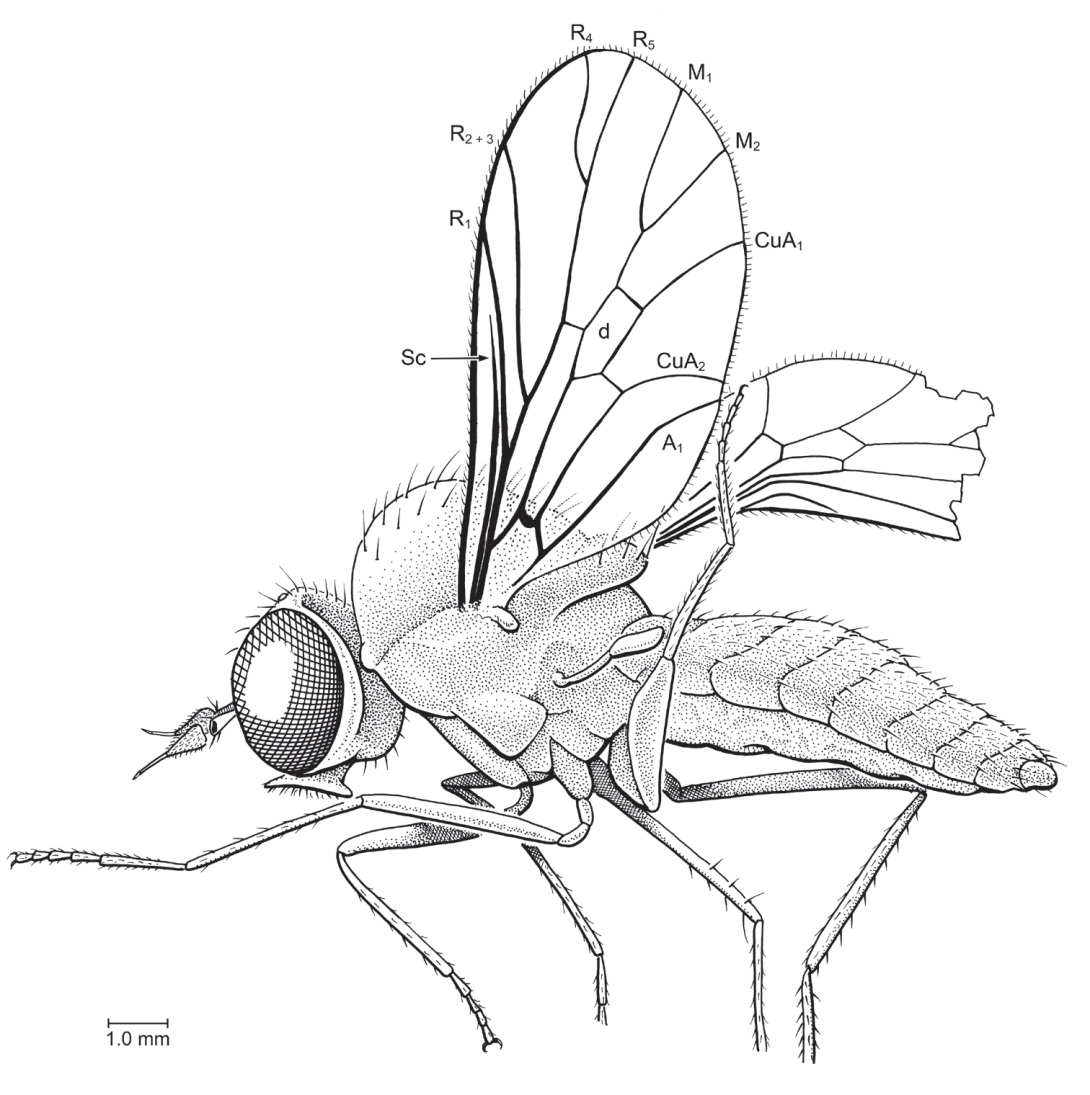
*Hilarimorphites burmanica* Grimaldi & Cumming, sp. n. (Apystomyiiidae) in Burmese amber, as preserved. Holotype, AMNH 098.

##### Type.

Holotype female, AMNH Bu-098, in amber from Myanmar: Kachin, Tanai Village (on Ledo Rd. ca. 105 km Myitkyna). Amber is a deep, clear yellow, 15 × 10 × 5 mm, and was embedded in epoxy and trimmed to a wedge shape in order to maximize a full lateral view of the fly and its venation. The piece also contains a male chironomid and a thrips (Thysanoptera).

##### Etymology.

From Burma (Myanmar).

##### Discussion.

*Hilarimorphites* was known only from Turonian-aged amber of central New Jersey, USA, and besides the new species in Burmese amber a very similar taxon is also now known from the Upper Jurassic of Kazakhstan. Mostovski (1999) described *Apystomimus zaitzevi*, preserved as a compression from the Karabastau Formation (Upper Jurassic) of the famous Karatau-Mikhailova Lagerstätte. That well-preserved specimen has a venation indistinguishable from that of *Hilarimorphites* Grimaldi & Cumming, 1999. *Apystomimus* differs from that genus by having small, brachypterous wings (ca. 0.5× length of the body) and very long cerci (nearly 0.5x length of wing; *Hilarimorphites* has very small cerci typical of lower Brachycera). Since these are autapomorphic features of *Apystomimus*, it could be appropriate to synonymize one of the genera (although *Apystomimus* is more aptly named, *Hilarimorphites* has date precedence by two months). *Hilarimorphites* was originally placed in the Hilarimorphidae, and Mostovski placed *Apystomimus* in Asilomorpha family-incertae sedis (but near the extant genus *Apystomyia*). Recent *Hilarimorpha* lack the discal cell, they have the cup cell closed, and lack a well-developed anal lobe while retaining a vestige of the anal vein, so the venation of the fossils is far more easily derived from *Apystomyia*. Thus, we agree that *Hilarimorphites* and *Apystomima* should both be classified in Apystomyiidae. The wing of *Hilarimorphites* differs from that of *Apystomyia* by the following: slightly shorter R_1_ and R_2+3_ veins; fork of R_4_+R_5_ less divergent, the branches slightly longer; cells br and bm significantly larger; cell cup significantly larger, with veins CuA_2_ and CuP meeting just before or at the wing margin, or not all (vs. CuA_2_+CuP with a long stem in *Apystomyia*); anal lobe of wing not protruding; and cell d much shorter, its length ca. 3× the width in *Hilarimorphites* (vs. 5 × the width in *Apystomyia*).

*Hilarimorphites burmanica* is intermediate in age between the previously known fossils, and greatly extends the geographic range. An extinct clade or grade of Apystomyiidae occurred minimally throughout Laurasia from the Upper Jurassic to the Upper Cretaceous, which is an age that is consistent with its hypothesized sister-group relationship near Eremoneura (Grimaldi and Cumming, 1999; Grimaldi and Engel, 2005; Wiegmann et al., 2011). Oddly, there are no other fossils as yet known of the family, not even from prolific and diverse Tertiary deposits like Baltic amber.

### Family Tethepomyiidae

**Diagnosis.** Small flies 1.5 mm in total body length, with venation and other features of the wing reduced. Vein CuA_1_-CuA_2_ comprised of short fork; vein M simple; vein A either absent or reduced to vestige at base of wings. Eyes very large, extensively holoptic in males. Cervical region long, head well separated from thorax; mesonotum compact, scutellum very short.

This is a highly specialized family of Diptera known only in amber from the Cretaceous of New Jersey, USA (Grimaldi and Cumming, 1999), Spain (Grimaldi and Arillo 2008), and now Myanmar (herein), comprised of two genera, *Tethepomyia* Grimaldi & Cumming and *Tethepomima* Grimaldi & Arillo. Discovery of the female of a new species in Burmese amber reveals the oviscapt to be a highly specialized, aculeus-type possibly used for parasitizing insect hosts. A few structures of tethepomyiids are similar to that of the Cretaceous brachyceran family Eremochaetidae (Ussatchev 1968; Kovalev 1986, 1989; Ren and Guo 1995; Mostovski 1997), which are discussed by (Grimaldi and Arillo 2008: p. 264). The aculeate oviscapt is now known to be another shared similarity (below). Either the highly reduced venation of tethepomyiids is a result of miniaturization, or these two families are unrelated and share remarkably convergent features. Tethepomyiidae were originally considered to possibly be nematoceran (Grimaldi and Cumming 1999), but we are including the family in this report since the oviscapt reveals a likely close relationship with Eremochaetidae, which are definitive Brachycera.

#### Genus Tethepomyia

urn:lsid:zoobank.org:act:BAEACD0A-8879-4761-95DB-4AF8297CADA0

http://species-id.net/wiki/Hilarimorphites_burmanica

http://species-id.net/wiki/Tethepomyia

Tethepomyia Grimaldi and Cumming 1999: 6. By original designation.

##### Diagnosis

(emended). Distinguished from *Tethepomima* by the following: Most or all of antennal flagellum lost; mesonotum bare, devoid of setae or setulae; apical tibial spurs absent; costal vein incomplete, not reaching to apex of Rs; costal spinules and fringe of fine setae on posterior margin of wing lost; alula and anal lobe lost; veins R_2+3_ and R_4+5_ lost (Rs simple, unbranched), crossvein r-m lost.

##### Type Species.

*Tethepomyia thauma* Grimaldi and Cumming 1999: 6. By original designation.

#### Tethepomyia zigrasi

Grimaldi & Arillo sp. n.

urn:lsid:zoobank.org:act:1D051ABF-34FA-461F-8583-F3E6CB322FCF

http://species-id.net/wiki/Tethepomyia_zigrasi

Fig. 10

##### Diagnosis.

Distinguished from the other two species of the genus, which are known only from males (*Tethepomyia thauma* Grimaldi and Cumming: New Jersey amber; and *Tethepomyia buruhandi* Grimaldi and Arillo: Spanish amber), by the following: thickened costal and Rs veins, bases of M and Cu complete; dorsoventral differentiation of eye facets (in female, undoubtedly more differentiated in males); U-shaped basal flagellomere large, pedicel small, indistinct. Known only from female.

##### Description.

Body length (tip of basal flagellomere to posterior-most surface of tergite VIII) 2.15 mm. HEAD: Hemispherical in female; eyes very large, covering most of head, only small strip of gena exposed [view of face and frons not visible]. Dorsal eyes facets approximately 0.5× diameter of ventral facet; eye completely bare, no interfacetal setulae. No setae apparent on gena or frons. Ocelli possibly on small tubercles – small, digitate lobes in this area [but details obscure]. Antenna with large, crescent-shaped basal flagellomere; pedicel apparently small [indistinct]. Proboscis and palps not visible [ventral surface of head covered with bubble]. Posterior surface of head evenly and shallowly concave. Cervical region long; head not adpressed to pronotum.

*Thorax*: Small and short, L = 0.55 mm, with scutum arched, posterior half long and sloped; scutum and scutellum devoid of acrostichals or setae. Scutellum short, length ca. 0.20 × that of scutum; posterior margin flat and slightly concave, not acute. Legs bare, devoid of setae or setulae; with femora slightly swollen in middle. Fore and mid coxae adjacent, hind tibia with coxal-trochanteral articulation facing anteriad (hind legs apparently held forward). Tibiae long and slender (slightly shorter than respective femur). Metatibia slightly bowed, as if to fit tightly against ventral surface of femur. Tarsi short, with basitarsomere only slightly longer than tarsomere 2 [most tarsomeres obscured by layer of air). Halter long; knob large, length of stem approximately 2.2 × greatest diameter of knob; stem without setae. Forewing with reduced venation; veins extremely light (particularly M and Cu); microtrichia of forewing either absent or so microscopic as to not be visible; no costal spinules or fringe of fine setulae on posterior margin of wing. Vein C short, extended to only ca. 0.6 × length of wing, sclerotized, swollen towards apex; apex of R_1_ fused with swollen portion of C. Rs thick, width slightly increased apicad; vein incomplete, not reaching wing margin/tip. Vein M faint, complete, tip evanescent and not reaching wing margin. Vein CuA faint, with short fork CuA_1_–CuA_2_ (length of fork 0.7 × length of stem); branches of fork curved towards anal region. What appears as deep fold (CuP?) parallel and posterior to stem of CuA. Faint, short vein A at base of posterior portion of wing; anal lobe and alula not present.

*Abdomen*: Tergites and sternites well developed, sclerotized; segments I – VI short (I longest), tergite VII long, sclerotized, length approximately equal to that of tII through tVI, with deeply incised membranous region basally, dorsal and lateral surfaces concave. Sternite VII very large, lobe-like, suspended beneath abdomen; apex pointed, bearing three short, sharp spines. Base of tVII apparently articulating with apex of tVI; tVII+VIII formed into a curved, sclerotized, sharp ovipositor-like structure, with a small, sharp, sclerotized spine at tip. Spine at tip of abdomen/oviscapt (sts IX) apparently interdigitating between three spines of sVII.

**Figure 10.**
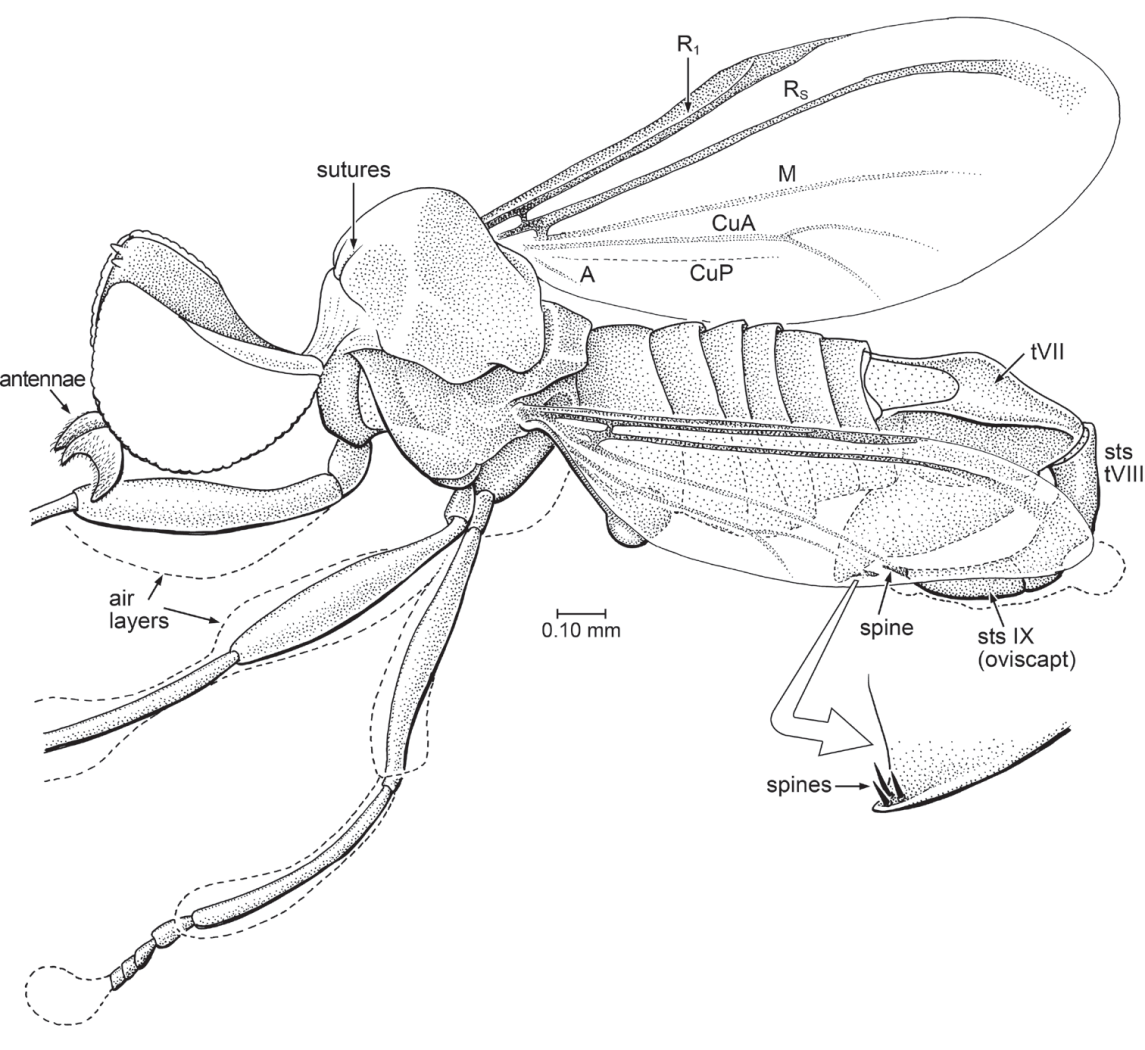
*Tethepomyia zigrasi* Grimaldi & Arillo, sp. n. (Tethepomyiidae) in Burmese amber, also showing ventral detail of distal portion of abdomen. Private collection of James Zigras. sts: syntergosternite.

##### Type.

Holotype, Female: Myanmar, Kachin State, Early Cenomanian. Specimen is in excellent condition and is in the private collection of James Zigras.

##### Etymology.

Patronym for James Zigras, for allowing preparation and study of this remarkable specimen.

##### Discussion.

*Tethepomyia zigrasi* sp. n. appears to be a sister group to *Tethepomyia buruhandi* + *thauma*, from Spanish and New Jersey ambers, respectively. *Tethepomyia zigrasi* retains the bases of M, Rs, and Cu, which the other two species have lost. It shares with *Tethepomyia buruhandi* and *Tethepomyia thauma* many losses: of the antennal stylus, tibial spurs, crossvein r-m, veins R_2+3_ and R_4+5_, costal vestiture, fringe of marginal setulae on the wing, as well as the reduction of vein C.

With little question the oviscapt of *Tethepomyia zigrasi* is a hypodermic-like (“aculeus”) structure, probably used for injecting its eggs into hosts. It was probably a parasitoid. An oviscapt of similar specialization has sporadically evolved in Diptera. It occurs in a few Phoridae (e.g., *Apocephalus*), all Pipunculidae, and within the Schizophora in some Conopidae (e.g., *Stylogaster*), most Tephritoidea, all Cryptochaetidae, and probably other families. The trait appears to have evolved most often in parasitoid groups (all those listed above except tephritoids). Most tephritoids inject their eggs into fruits or stems, though a few (like Pyrgotidae) are parasitoids. Fine structure of the injecting oviscapt reveals its convergent development: what is labelled as the “ovipositor” in Pipunculidae (Hardy, 1989: 747) is probably a sclerotized, spine-like derivative of the cercus. In Cryptochaetidae the syringe-like oviscapt is sternite VIII; in Tephritoidea the oviscapt is a telescoping structure composed of segments 7–9. Interestingly, *Tethepomyia* has a suite of other convergent features similar to those of parasitoid families. Like Pipunculidae, Tethepomyiidae possesse large eyes; like Pipunculidae and Cryptochaetidae the family has large pulvilli; and like Cryptochaetidae the basal flagellomere is enlarged and the arista minute to lost. These are probably functionally correlated features.

### Unplaced to Family

#### Myanmyia

Grimaldi gen. n.

urn:lsid:zoobank.org:act:7090815B-8878-487D-9980-78581DB36111

http://species-id.net/wiki/Myanmyia

##### Diagnosis.

Distinctive small flies (body length less than 1.5 mm) with antennal stylus arista-like and terminal, having a single article; face without ptilinal suture; median margins of eyes very close on frons; maxillary palpus two-segmented; mesonotum with dorsocentral and scutellar setae; wing venation highly reduced, with R_2+3_ and R_4+5_ each unbranched, M unbranched and evanescent at both ends, Cu simple; female with pair of long, digitate, unsegmented cerci.

##### Etymology.

From Myanmar, country of origin, and –*myia*, a common suffix referring to the feminine Greek word for fly.

##### Type Species.

*Myanmyia asteiformia* sp. n. By present designation.

##### Discussion.

This is a perplexing little fly. Chaetotaxy of the thorax, the wing venation, and even body shape are strikingly similar to acalyptrate flies in the Asteiidae. Convergent wing features of the two groups include short R_1_ and R_2+3_ veins; a straight R_4+5_ that meets the tip of the wing, and even microtrichia that are arranged in rows. However, *Myanmyia* is not even a cyclorrhaphan, by virtue of the terminal (versus dorsal) arista-like stylus, lack of a ptilinum, and presence of two-segmented (vs. 1-segmented) palpi. With the exception of a few very basal Recent and extinct Platypezidae, almost all other Cyclorrhapha have a dorsal arista. Two-segmented palpi exclude *Myanmyia* from the Eremoneura (the apparent basal segment of the two segmented palpi seen in some Phoridae is probably a palpifer [Cumming and Wood 2009]). While some empidoids (e.g., Cretaceous *Nemedina* genus-group species [Grimaldi and Cumming 1999]) have short R veins and faint M and Cu veins, the branching pattern for these flies differs significantly at the base from that of *Myanmyia*.

#### Myanmyia asteiformia

Grimaldi sp. n.

urn:lsid:zoobank.org:act:3BD4F73D-375A-4B7F-8637-5449BC695495

http://species-id.net/wiki/Myanmyia_asteiformia

Fig. 11

##### Diagnosis.

As for genus.

##### Description.

Body size small, length 1.35 mm (excluding antennae and cerci), slender. Wing length 1.05 mm. *Head*: Slightly wider than thorax [possibly preservational, as head is slightly compressed]. Antenna with cup-like pedicel, distal edge rimmed with fine, stiff setae; basal flagellomere drop-shaped, width approximately equal to length; arista-like stylus terminal, setulose, 1-articled (no small basal articles), length approximately 3 × length of basal flagellomere. Eyes large (occupying virtually entire lateral surface of head), bare, with slight dorsoventral differentiation of facets (dorsal facets ca. 2 × diameter of ventral ones); inner margins of eyes (on frons) very close, width of separation equal to ca. 3 facet diameters. Ptilinal suture absent. Maxillary palp 2-segmented, with apical segment clavate and basal segment slender. Labrum long, very slender; hypopharynx (?) stylet-like; labellum small. Gena very shallow or barely developed (not apparent). Postocciput broad, concave. *Thorax*: Slender, with the following dorsal setae (*per side*): 1 postpronotal, 4 supra-alar/notopleurals, 3 postsutural dorsocentrals (posterior one largest), 2 pairs scutellars [pleura not visible]. Legs of moderate length, setulose, without distinctive spines/spurs. Pretarsus with claws well developed, but no pulvilli. Wing: Long, slender, W/L = 0.33; membrane microtrichia arranged in oblique rows (between R veins) and longitudinal rows (portions of space between R_4+5_ and M). Vein C slightly beyond apex of R_4+5_, no humeral or subcostal breaks; with long, sparse spinules. Sc short, very faint [best seen when tilting specimen]. Base of vein R thick, R_1_ short (length 0.3 × length of wing); R_2+3_ unbranched, meeting C at ⅔ the length of wing. R_4+5_ straight, extended to tip of wing, unbranched. Vein M simple, unbranched, very lightly sclerotized; both ends evanescent. Sc. Vein A thick, heavily sclerotized strip along alular edge of wing. Anal lobe and alula not developed. Halter: with large, darkened knob, stem approximately same length as knob or slightly longer. *Abdomen*: Tergites I—VII well developed, with sparse setulae, without macrosetae; sternites II, III, IV large, bare; segment V is tubular; VI, VII ring-like; terminal segment bearing pair of long, finger-like, one-segmented cerci. Presence/absence of abdominal muscle plaques not visible.

**Figure 11.**
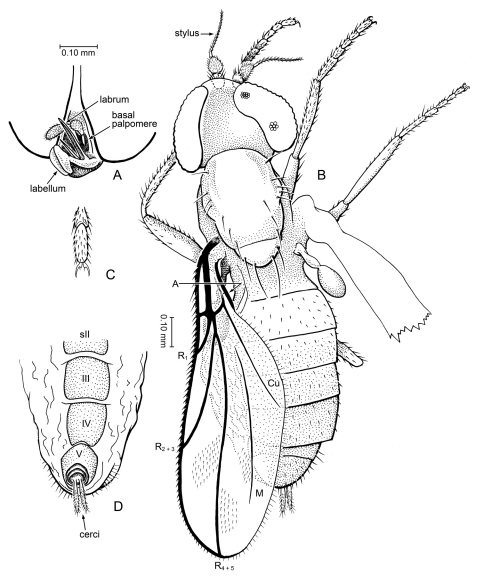
**a-d** *Myanmyia asteiformia* Grimaldi, gen. et sp. n. (unplaced to family), in Burmese amber (holotype, AMNH Bu1616) **a** Anteroventral detail of head **b** dorsal habitus, as preserved **c** pretarsus. **d** apex of abdomen, ventral view.

##### Type.

Holotype, female, AMNH Bu1616, in amber from northern Myanmar: Kachin State, Tanai Village, 105 km NW Mytikyina. The holotype is the sole inclusion in a clear amber-colored piece 9 × 6 × 2 mm. Ventral surface of the thorax and the abdomen are compressed, and a crack through the thorax obscures some details. The left wing of the unique specimen is well preserved, but venation is optimally observed by tilting and observing the piece at various oblique angles. Right wing is twisted, but in oblique view additional details of venation are visible.

##### Etymology.

L., for like, and *Asteia* (type genus of the Asteiidae, a family of Schizophoran flies) and Latin –*formia*, meaning like, in reference to the similarity of the unrelated two taxa in body shape, size, and wing venation.

## Supplementary Material

XML Treatment for Cretaceogaster

XML Treatment for Lysistrata

XML Treatment for Lysistrata emerita

XML Treatment for Cretoxyla

XML Treatment for Cretoxyla azari

XML Treatment for Xylomyidae

XML Treatment for Cratotabanus

XML Treatment for Cratotabanus newjerseyensis

XML Treatment for Schlingeromyia

XML Treatment for Schlingeromyia minuta

XML Treatment for Burmacyrtus

XML Treatment for Burmacyrtus rusmithi

XML Treatment for Microburmyia

XML Treatment for Microburmyia analvena

XML Treatment for Microburmyia veanalvena

XML Treatment for Kumaromyia

XML Treatment for Kumaromyia burmitica

XML Treatment for Hilarimorphites burmanica

XML Treatment for Tethepomyia

XML Treatment for Tethepomyia zigrasi

XML Treatment for Myanmyia

XML Treatment for Myanmyia asteiformia

## References

[B1] Alonso J Arillo A Barrón E Corral JC Grimalt J López JF López R Martínez-Delclòs X Ortuño VM Peñalver E Trincão PR (2000) A new fossil resin with biological inclusions in Lower Cretaceous deposits from Álava (Northern Spain, Basque-Cantabrian Basin). Journal of Paleontology 74 (1): 158-178.

[B2] Chandler PJ (2010) (Ed) *A Dipterist’s Handbook, 2nd Edition*. The Amateur Entomologist, vol. 15. The Amateur Entomologist’s Society, Kent UK: 525 pp. + 32 plates.

[B3] Corral, JC López Del Valle R Alonso J (1999) El ámbar cretácico de Álava (Cuenca Vasco Cantábrica, norte de España). Su colecta y preparación. Estudias Museo Ciencias de Naturales Álava 14: 7-21

[B4] Cruickshank RD Ko K (2003) Geology of an amber locality in the Hukawng Valley, northern Myanmar. Journal of Asian Earth Sciences 21: 441-455. 10.1016/S1367-9120(02)00044-5

[B5] Cumming JM Wood DM (2009) Adult morphology and terminology. In: Brown BV et al. (Eds) Manual of Central American Diptera, vol. 1.Ottawa: NRC Research Press, 9–50.

[B6] Delclòs X Arillo A Peñalver E Barrón E Soriano C López del Valle R Bernárdez E Corral C Ortuño VM (2007) Fossiliferous amber deposits from the Cretaceous (Albian) of Spain. Comptes Rendus Palevol 6: 135-149. 10.1016/j.crpv.2006.09.003

[B7] Evenhuis NL (1994) Catalogue of the Fossil Flies of the World (Insecta: Diptera). Leiden: Backhuys, 600 pp.

[B8] Evenhuis NL (2002) Review of the Tertiary microbombyliids (Diptera: Mythicomyiidae) in Baltic, Bitterfeld, and Dominican amber. Zootaxa 100: 1-15.

[B9] Gaimari S Mostovski MB (2000) *Burmapsilocephala cockerelli*, a new genus and species of Asiloidea (Diptera) from Burmese amber. Bulletin of the Natural History Museum, London (Geology) 56: 43-45.

[B10] Grimaldi D (1995) A remarkable new species of *Ogcodes* (Diptera: Acroceridae) in Dominican amber. American Museum Novitates 3127: 1-8.

[B11] Grimaldi D (1999) The co-radiations of pollinating insects and angiosperms in the Cretaceous. Annals of the Missouri Botanical Garden 86: 373-406. 10.2307/2666181

[B12] Grimaldi D Arillo A (2008) The Tethepomyiidae, a new family of enigmatic Cretaceous Diptera. Alavesia 2: 259-265.

[B13] Grimaldi DA Cumming JM (1999) Brachyceran Diptera in Cretaceous ambers and Mesozoic diversification of the Eremoneura. Bulletin of the American Museum of Natural History 239: 1-124.

[B14] Grimaldi DA Cumming JM Arillo A (2009) Chimeromyiidae, a new family of Eremoneuran Diptera from the Cretaceous. Zootaxa 2078: 34-54.

[B15] Grimaldi DA Engel MS (2005) Evolution of the Insects. New York: Cambridge Univ. Press. 755 pp.

[B16] Grimaldi D Engel MS Nascimbene PC (2002) Fossiliferous Cretaceous amber from Myanmar (Burma): its rediscovery, biotic diversity, and paleontological significance. American Museum Novitates 3361: 1-72.

[B17] Hall JC (1981) Bombyliidae, In: McAlpine JF et al. (Eds) Manual of Nearctic Diptera, vol. 1. Ottawa: Research Branch Agriculture Canada Monograph 27, 589–602.

[B18] Hardy DE (1987) Pipunculidae. In: McAlpine JF et al. (Eds) Manual of Nearctic Diptera, vol. 2. Ottawa: Research Branch Agriculture Canada Monograph 28, 745–748.

[B19] Hauser M (2007) Baltic amber Therevidae and Apsilocephalidae (Diptera). Studia Dipterologica 14: 37-59.

[B20] Hauser M Irwin ME (2005) Fossil Therevidae (Insecta: Diptera) from Florissant, Colorado (Upper Eocene). Journal of Systematic Palaeontology 3: 393-401. 10.1017/S1477201905001690

[B21] Hauser M Winterton SL (2007) A new fossil genus of small-headed flies (Diptera: Acroceridae: Philopotinae) from Baltic amber. Annals of the Entomological Society of America 100: 152-156. 10.1603/0013-8746(2007)100[152:ANFGOS]2.0.CO;2

[B22] Huang DY Lin QB (2007) A new soldier fly (Diptera, Stratiomyidae) from the Lower Cretaceous of Liaoning Province, northeast China. Cretaceous Research 28: 317-321. 10.1016/j.cretres.2006.05.006

[B23] Kovalev VG (1986) Infraorders Bibionomorpha and Asilomorpha. In: Insects in the Early Cretaceous Ecosystems of Western Mongolia. Trudy Sovmestnoy Sovestsko-Mongol’skoy Paleontologischeskii Espedidtsii 28, 125–154. [In Russian].

[B24] Kovalev VG (1989) Bremochaetidae [sic: Eremochaetidae], the Mesozoic family of brachycerous dipterans. Paleontological Journal 23: 100-105.

[B25] Martill DM Bechly G Loveridge RF (2007) (Eds) The Crato Fossil Beds of Brazil. Window into An Ancient World. Cambridge: Cambridge University Press, 625 pp.

[B26] Martins-Neto RG (2003) The fossil tabanids (Diptera Tabanidae): When they began to appreciate warm blood and why they began transmit [sic] diseases? Memórias Instituto Oswaldo Cruz 98 (suppl. 1): 29–34. 10.1590/S0074-02762003000900006 12687759

[B27] Martins-Neto RG Kucero-Santos JC (1994) Um novo gênero e uma nova espécie de Mutuca (Insecta, Diptera, Tabanidae) da Formação Santana (Cretáceo Inferior). Bacia do Araripe, Nordeste do Brasil. Acta Geologica Leopoldensia 39: 289-297.

[B28] Mazzarolo LA Amorim DS (2000) *Cratomyia macrorrhyncha*, a Lower Cretaceous brachyceran fossil from the Santana Formation, Brazil, representing a new species, genus and family of the Stratiomyomorpha (Diptera). Insect Systematics and Evolution 31: 91-102. 10.1163/187631200X00336

[B29] Mostovski MB (1997) To the knowledge of Archisargoidea (Diptera, Brachycera). Families Eremochaetidae and Archisargidae. Russian Entomological Journal 5: 117-124.

[B30] Mostovski MB (1998) A brief review of brachycerous flies (Diptera, Brachycera) in the Mesozoic, with descriptions of some curious taxa. AMBA, Proc. 1^st^ International Palaeoentomological Conference, Moscow, 103–110.

[B31] Mostovski MB (1999) On an interesting find of a brachycerous fly (Diptera, Brachycera) in the Jurassic of Kazakhstan. Paleontological Journal 33: 406-408.

[B32] Mostovski MB Jarzembowski EA Coram RA (2003) Horseflies and athericids (Diptera: Tabanidae, Athericidae) from the Lower Cretaceous of England and Transbaikalia. Paleontological Journal 37: 162-169.

[B33] Nagatomi A (1996) An essay on phylogeny of the orthorrhaphous Brachycera (Diptera). Entomologist’s Monthly Magazine 132: 95-148.

[B34] Nagatomi A Liu N (1994) Apystomyiidae, a new family of Asiloidea (Diptera). Acta Zoologica Academiae Scientiarum Hungaricae 40: 203-218.

[B35] Nagatomi A Yang D (1998) A review of extinct Mesozoic genera and families of Brachycera (Insecta, Diptera, Orthorrhapha). Entomologists’ Monthly Magazine 134: 95-192.

[B36] Nascimbene PC Silverstein H (2000) The preparation of fragile Cretaceous ambers for conservation and study of organismal inclusions. In: Grimaldi D (Ed.). , Studies on Fossil in Amber, with Particular Reference to the Cretaceous of New Jersey. Leiden: Backhuys Publishers: 93-102.

[B37] O’Hara JE (2011) Cyber nomenclaturalists and the “CESA itch”. Zootaxa 2933: 57-64.

[B38] Peñalver E Delclòs X (2010) Spanish amber. In: Penney D (Ed.). , Biodiversity of Fossils in Amber from the Major World Deposits. Siri Scientific Press, Manchester, UK: 236-270.

[B39] Pike EM (1995) Amber Taphonomy and the Grassy Lake, Alberta, Amber Fauna. Ph.D. thesis, University of Calgary, Alberta.

[B40] Ren D (1998) Late Jurassic Brachycera from northeastern China. Acta Zootaxonomica Sinica 23: 65-83.

[B41] Ren D Guo Z (1995) A new genus and two new species of short-horned flies of Upper Jurassic from northeast China (Diptera: Eremochaetidae). Entomologia Sinica 2: 300-307.

[B42] Röder G (1986) Zur Morphologie des Prätarsus der Diptera und Mecoptera. Zoologische Jahrbücher. Abteilung für Anatomie und Ontogenie der Tiere 144 (4): 465-502.

[B43] Sinclair BJ Cumming JM Wood DM (1994) Homology and phylogenetic implications of male genitalia in Diptera – Lower Brachycera. Entomologica Scandinavica 24: 407-432. 10.1163/187631293X00190

[B44] Teskey HJ (1971) A new soldier fly from Canadian amber (Diptera: Stratiomyidae). The Canadian Entomologist 103: 1659-1661. 10.4039/Ent1031659-12

[B45] Trautwein MD Wiegmann BM Yeates DK (2010) A multigene phylogeny of the fly superfamily Asiloidea (Insecta): Taxon sampling and additional genes reveal the sister-group to all higher flies (Cyclorrhapha). Molecular Phylogenetics and Evolution 56: 918-930. 10.1016/j.ympev.2010.04.017 20399874

[B46] Ussatchev DA (1968) New Jurassic Asilomorpha (Diptera) in Karatau. Entomologicheskoe Obozrenie 47: 617–628. [English translation in Entomological Review 47: 378–384].

[B47] Whalley PES Jarzembowski EA (1985) Fossil insects from the lithographic limestone of Montsec (Late Jurassic – Early Cretaceous), Lérida Province, Spain. Bulletin of the British Museum (Natural History) (Geology) 38: 381-412.

[B48] Wiegmann BM Mitter C Thompson FC (1993) Evolutionary origin of the Cyclorrhapha (Diptera): tests of alternative morphological hypotheses. Cladistics 9: 41-81. 10.1111/j.1096-0031.1993.tb00208.x 34929938

[B49] Wiegmann BM Trautwin MD Winkler IS (+ 24 authors) (2011) Episodic radiations in the fly tree of life. Proceedings of the National Academy of Sciences, USA 108 (14): 5690-5695. 10.1073/pnas.1012675108 PMC3078341 21402926

[B50] Winterton SL Irwin ME (2008) *Kaurimyia* gen. nov.: discovery of Apsilocephalidae (Diptera: therevoid clade) in New Zealand. Zootaxa 1779: 38-44.

[B51] Winterton SL Wiegmann BM Schlinger EI (2007) Phylogeny and Bayesian divergence time estimations of small-headed flies (Diptera: Acroceridae) using multiple molecular markers. Molecular Phylogenetics and Evolution 43 (3): 808-832. 10.1016/j.ympev.2006.08.015 17196837

[B52] Woodley NE (1986) Parhadrestiinae, a new subfamily for *Parhadrestia* James and *Cretaceogaster* Teskey (Diptera: Stratiomyidae). Systematic Entomology 11: 377-387. 10.1111/j.1365-3113.1986.tb00189.x

[B53] Woodley NE (1989) Phylogeny and classification of the “orthorrhaphous” Brachycera. In McAlpine JF et al. (Eds), Manual of Nearctic Diptera, vol. 3. Research Agriculture: Ottawa, 1371–1395.

[B54] Woodley NE (2001) A world catalogue of Stratiomyidae (Insecta: Diptera). Myia 11: viii + 475 pp.

[B55] Woodley NE Borkent A Wheeler TA (2009) Phylogeny of the Diptera. In: Brown BV et al. (Eds), Manual of Central American Diptera, vol. 1.NRC Research Press, Ottawa, 79–94.

[B56] Yeates DK (1994) Cladistics and classification of the Bombyliidae (Diptera: Asiloidea). Bulletin of the American Museum of Natural History 219: 191 pp.

[B57] Yeates DK (2002) Relationships of extant lower Brachycera (Diptera): a quantitative synthesis of morphological characters. Zoologica Scripta 31: 105-121. 10.1046/j.0300-3256.2001.00077.x

[B58] Yeates DK Irwin ME Wiegmann BM (2003) Ocoidae, a new family of asiloid flies (Diptera: Brachycera: Asiloidea), based on *Ocoa chilensis* gen. and sp. n. from Chile, South America. Systematic Entomology 28: 417-431. 10.1046/j.1365-3113.2003.00224.x

[B59] Yeates DK Irwin ME Wiegmann BM (2006) Evocoidae (Diptera: Asiloidea), a new family name for Ocoidae, based on *Evocoa*, a replacement name for the Chilean genus *Ocoa* Yeates, Irwin, and Wiegmann 2003. Systematic Entomology 31: 373. 10.1111/j.1365-3113.2006.00332.x

[B60] Yeates DK Wiegmann BM Courtney GW Meier R Lambkin C Pape T (2007) Phylogeny and systematics of Diptera: Two decades of progress and prospects. Zootaxa 1668: 565-590.

[B61] Zhang JF Zhang S Li LY (1993) Mesozoic gadflies (Insecta: Diptera). Acta Palaeontologica Sinica 32: 662-672.

[B62] Zhang KY (2009) Systematics of Mesozoic Brachycera from China (Insecta: Diptera). Ph.D. Dissertation, China Agricultural University. 183 pp.

[B63] Zherikhin VV Sukatsheva ID (1973) On Cretaceous insects from “amber” (retinites) of northern Siberia. In: 24^th^ Annual Report of Lectures in Memory of N. A. Kolodovsky, Nauka, Moscow 4–48.

